# Aryloxyalkanoic Acids as Non-Covalent Modifiers of the Allosteric Properties of Hemoglobin

**DOI:** 10.3390/molecules21081057

**Published:** 2016-08-13

**Authors:** Abdelsattar M. Omar, Mona A. Mahran, Mohini S. Ghatge, Faida H. A. Bamane, Mostafa H. Ahmed, Moustafa E. El-Araby, Osheiza Abdulmalik, Martin K. Safo

**Affiliations:** 1 Department of Pharmaceutical Chemistry, Faculty of Pharmacy, King Abdulaziz University, Alsulaymanyah, Jeddah 21589, Saudi Arabia; alamka@gmail.com; 2 Department of Pharmaceutical Chemistry, Faculty of Pharmacy, Al-Azhar University, Cairo 11884, Egypt; 3 Department of Pharmaceutical Chemistry, Faculty of Pharmacy, Alexandria University, Alexandria 21521, Egypt; mahranmona@yahoo.com; 4 Department of Medicinal Chemistry, and The Institute for Structural Biology, Drug Discovery and Development, School of Pharmacy, Virginia Commonwealth University, Richmond, VA 23298, USA; msghatge@vcu.edu (M.S.G.); ahmedmh@vcu.edu (M.H.A.); 5 Department of Biochemistry, Faculty of Medicine, King Abdulaziz University, Alsulaymanyah, Jeddah 21589, Saudi Arabia; fbamane@yahoo.com; 6 Department of Pharmaceutical Organic Chemistry, Faculty of Pharmacy, Helwan University, Cairo 11790, Egypt; 7 Division of Hematology, The Children’s Hospital of Philadelphia, Philadelphia, PA 19104, USA; abdulmalik@email.chop.edu

**Keywords:** hemoglobin, sickle cell disease, aryloxyalkanoic acids, halogenated benzene, imidazole, antisickling, oxygen equilibrium curve, high affinity, low affinity, crystal structure

## Abstract

Hemoglobin (Hb) modifiers that stereospecifically inhibit sickle hemoglobin polymer formation and/or allosterically increase Hb affinity for oxygen have been shown to prevent the primary pathophysiology of sickle cell disease (SCD), specifically, Hb polymerization and red blood cell sickling. Several such compounds are currently being clinically studied for the treatment of SCD. Based on the previously reported non-covalent Hb binding characteristics of substituted aryloxyalkanoic acids that exhibited antisickling properties, we designed, synthesized and evaluated 18 new compounds (KAUS II series) for enhanced antisickling activities. Surprisingly, select test compounds showed no antisickling effects or promoted erythrocyte sickling. Additionally, the compounds showed no significant effect on Hb oxygen affinity (or in some cases, even decreased the affinity for oxygen). The X-ray structure of deoxygenated Hb in complex with a prototype compound, KAUS-23, revealed that the effector bound in the central water cavity of the protein, providing atomic level explanations for the observed functional and biological activities. Although the structural modification did not lead to the anticipated biological effects, the findings provide important direction for designing candidate antisickling agents, as well as a framework for novel Hb allosteric effectors that conversely, decrease the protein affinity for oxygen for potential therapeutic use for hypoxic- and/or ischemic-related diseases.

## 1. Introduction

In its function of transporting oxygen from the lungs to tissues, hemoglobin (Hb) alternates between tense or T-state (unliganded or deoxygenated Hb) and the relaxed or R-state (liganded or oxygenated Hb). The equilibrium between the two states can be regulated by synthetic allosteric effectors that bind to the central water cavity or the surface of the protein [1,2]. Stabilization of the R-state and/or destabilization of the T-state shifts Hb oxygen equilibrium curve (OEC) to the left, producing a high-affinity Hb that is known to be beneficial for sickle cell disease (SCD) therapy [1,2]. The converse, where a low-affinity Hb is produced with OEC right-shifting compounds that are also potentially useful for the treatment of hypoxic- and/or ischemic-related diseases is also true [1,2]. Under hypoxia sickle Hb (Hb S) polymerizes into long, rigid, and insoluble fibers causing the primary pathophysiology associated with SCD, which leads to RBC sickling, vaso-occlusion, painful crises, organ damage, oxidative stress, hemolysis of RBCs, decreased vascular NO bioavailability, inflammation, impaired microvascular blood flow, morbidity and even mortality [3,4,5,6]. The polymerization process is exacerbated by the low oxygen affinity of Hb S, presumably as a result of unusually high concentration of 2,3-diphosphoglycerate and/or sphingosine phosphate in sickle red blood cells (RBC) [7,8,9,10]. The polymer is initiated by a primary interaction between the pathological β2Val6 from one Hb S molecule and a hydrophobic acceptor pocket in the region of β1Ala70, β1Phe85 and β1Leu88 of another Hb molecule, and further stabilized by several secondary contacts between the Hb molecules [11,12,13,14,15]. Disruption or weakening of the secondary contacts is known to reduce Hb S polymerization and RBC sickling as demonstrated by a large number of naturally occurring mutations that are known to mitigate the severity of SCD [16,17].

There have been several efforts to develop pharmacological interventions that increase Hb oxygen affinity to prevent the hypoxia-induced Hb S polymerization and/or directly destabilize polymer contacts [1,2,10]. The most promising are covalent modifiers of Hb, with examples such as aromatic aldehydes [18,19,20,21], ethacrynic acid derivatives [22], isothiocynates [23], and thiols [24]. Nonetheless nonspecific binding to other proteins and the resulting toxic effects has precluded several of these covalent modifiers from being considered as therapeutic agents [10]. Abraham and co-workers in the eighties showed that halogenated benzene derivatives or substituted aryloxyalkanoic acids bind non-covalently to Hb at multiple Hb sites, including the central water cavity or the CD-corner or the αTrp14 binding pocket at the surface of the protein [25,26,27,28,29]. Those compounds that bind exclusively to the CD-corner or αTrp14 site, e.g., 3,4-(dichlorobenzyl)oxyacetic acid or *p*-(bromobenzyl)oxyacetic acid showed no significant effect on Hb oxygen binding properties, however they exhibited weak antisickling effect, which was speculated to be due to weakening or destabilization of the polymer contacts [25,26,27,28]. On the other hand, compounds, e.g., clofibrate and several of its analogs or derivatives that bind to the central water cavity decreased Hb affinity for oxygen due to stabilization of T-state Hb [25,26,27,28,29,30,31,32]. Although, compounds that decrease Hb affinity for oxygen are expected to promote hypoxia-induced sickling, clofibrate was reported to exhibit a weak antisickling effect, which was attributed to the compound also binding to the αTrp14 site beside the central water cavity to destabilize the polymer [27].

Even though the reported αTrp14 binders showed marginal antisickling effects, the αTrp14 site remains an attractive target for drug design, which we decided to investigate. Consequently, we have structurally modified the previously studied aryloxyalkanoic acid pharmacophore into two series of compounds, 4-(imidazole-2-carbonyl)phenoxyalkanoic acids (**7a**–**I**; KAUS-1 to KAUS-8) and 4-(imidazol-2-yl)phenoxyalkanoic acids (**13**, **17a**–**e**, **21a**–**c**; KAUS-20 to KAUS-27) that are expected to bind to the αTrp14 site. The compounds have been tested for their effect on both sickle RBC morphology and Hb oxygen binding properties. One of the compounds, KAUS-23 was co-crystallized with deoxygenated Hb and its binding mode studied to gain insight into the compound’s functional and biological activities.

## 2. Results and Discussion

### 2.1. Compounds Designed to Bind at the αTrp14 Site of Hemoglobin

**Figure 1:**
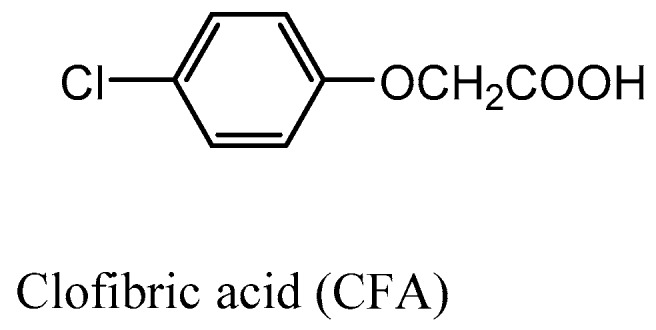
Example of previously studied aryloxyalkanoic acid derivatives.

Substituted aromatic compounds, e.g., aryloxyalkanoic acids, have been shown to bind non-covalently to Hb at the central water cavity or the surface of the protein, e.g., αTrp14 or CD-corner binding sites and display moderate or no antisickling effect, and in some instances even prosickling effects [22,23,24,25,29]. In most cases, the compounds that bind exclusively to the central water cavity showed no antisickling activity, due to stabilization of T-state Hb and the concomitant decrease in Hb affinity for oxygen [25,27,28]. In contrast, compounds that bind to the surface of the protein, e.g., the αTrp14 binding pocket exhibited moderate antisickling activities, which was attributed to direct polymer destabilization [25,26,27,28]. Other aryloxyalkanoic acids, e.g., clofibrate (Figure 1) which is known to bind to both the central water cavity and the αTrp14 site was reported to show both low-oxygen affinity and antisickling properties [27]. 

**Figure 2:**
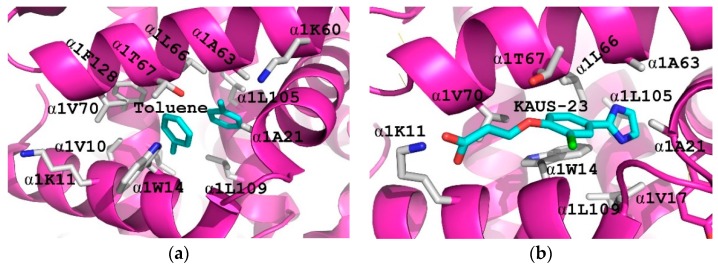
The αTrp14 binding pocket. (**a**) Binding of two molecules of toluene at the αTrp14 binding pocket of carbonmonoxy Hb (PDB ID: 1R1X). As noted in the text, CFA binds in a similar fashion but the coordinate is not available [27]; (**b**) Putative binding of KAUS-23 at the Trp14 binding pocket of deoxygenated Hb (PDB ID: 2DN2).

The αTrp14 binding pocket is lined with several hydrophobic residues, including αTrp14, αVal17, αAla21, αTyr24, αPhe128, αLeu105, αLeu109 and αLeu125, and hydrophilic residues at the mouth of the cavity including, αThr67, αLys11 and αLys60. The crystal structures of deoxygenated Hb in complex with 3,4-(dichlorobenzyl)oxyacetic acid or clofibrate or other aryloxyalkanoic acids that bind to the αTrp14 have been described previously [25,26,27,28,29]. Due to the low resolution nature of the structures, detailed interactions between the protein and compounds were not described, however the interactions with the surrounding hydrophobic residues were suggested to be predominantly hydrophobic and dipolar van der Waals in nature [25,26,27,28]. These compounds presumably bind similarly to the αTrp14 pocket like the toluene molecule whose coordinates are available from a more recently determined high-resolution structure (PDB code 1R1X)[33]. In this structure, the αTrp14 pocket shows two bound toluene molecules making hydrophobic interactions with αTrp14, αVal17, αTyr24, αThr67, αLue105, αLeu109, αLeu125, αPhe128, αVal10, αVal70 and αLeu66 (Figure 2a).

Based on the αTrp14 binding interactions with 3,4-[(dichlorobenzyl)oxy]acetic acid or clofibrate and the fact that two toluene molecules are able to fit the binding cavity (Figure 2a), we hypothesized that increasing the size of the aryloxyalkanoic acids for additional hydrophobic interactions with the αTrp14 pocket residues might lead to stronger compound binding, resulting in increased Hb affinity for oxygen and antisickling potency. We therefore embarked on a series of structural modifications of the aryloxyalkanoic acid pharmacophore by introducing an imidazole to the aryloxyalkanoic ring that we anticipate will increase hydrophobic interaction with the αTrp14 non-polar pocket residues as putatively illustrated in Figure 2b (please see Supplementary Material for a description of the docking process).

**Scheme 1:**
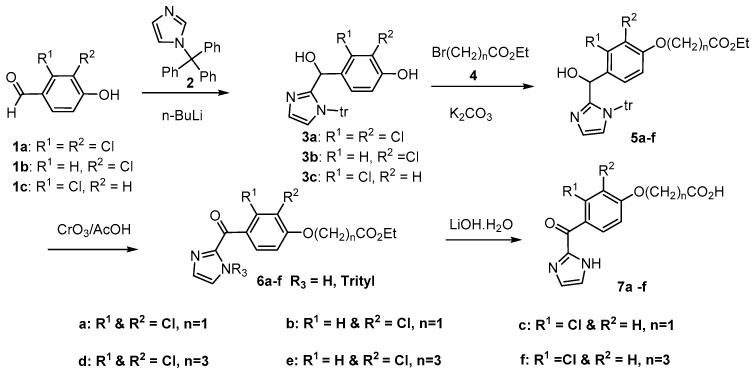
Synthesis of {[2,3-Di/mono substituted-4-(1*H*-imidazol-2yl)carbonyl]-phenoxy}acetic/butyric acids.

**Scheme 2:**
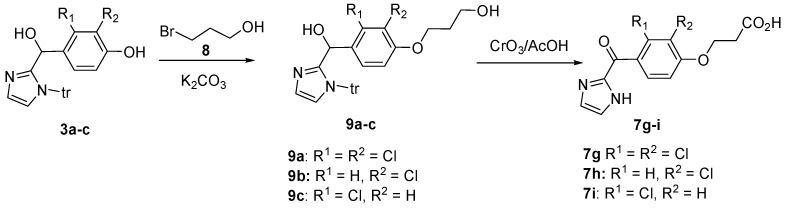
Synthesis of {[2,3-Di/mono substituted-4-(1*H*-imidazol-2yl)carbonyl]-phenoxy}propionic acids.

**Scheme 3:**
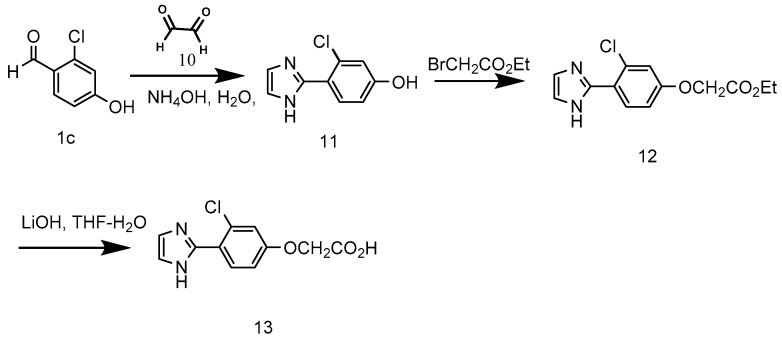
Synthesis of 2-[3-chloro-4-(1*H*-imidazol-2-yl)phenoxy]acetic acid.

**Scheme 4:**
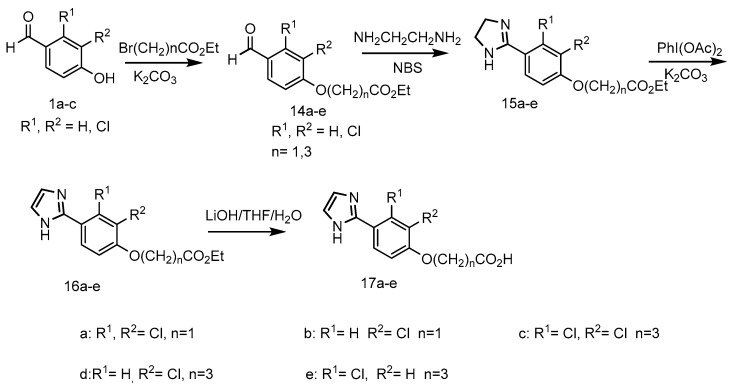
Synthesis of 2-[Di/monochloro-4-(1*H*-imidazol-2-yl)phenoxy]acetic/butyric acids.

**Scheme 5:**
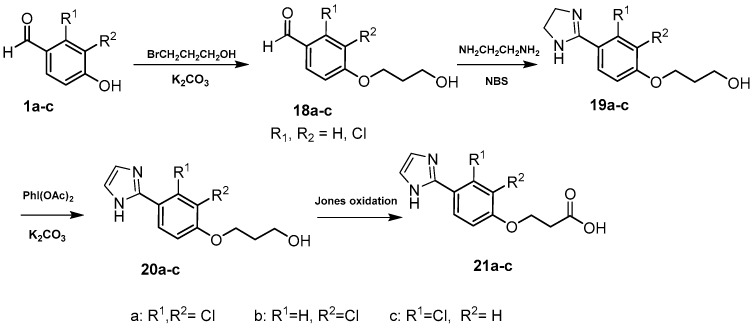
Synthesis of 2-[Di/monochloro-4-(1*H*-imidazol-2-yl)phenoxy]propionic acids.

Two classes of compounds, (imidazole-2-carbonyl)phenoxyalkanoic acids (**7a**–**I**; KAUS-1 to KAUS-8) and (imidazol-2-yl)phenoxyalkanoic acids (**13**, **17a**–**e**, **21a**–**c**; KAUS-20 to KAUS-27) were synthesized. The proposed compounds, like 3,4-(dichlorobenzyl)oxyacetic acid and clofibrate also contain carboxylate moieties that could potentially form salt-bridge/hydrogen-bond interactions with αLys60 and/or αThr67 located at the mouth of the pocket (Figure 2b). We should point out that there is no indication to suggest that the carboxylate of 3,4-(dichlorobenzyl)oxyacetic acid or clofibrate form such polar interactions. It was also anticipated that the compounds will exhibit minimal toxicity effect due to their non-covalent interactions with Hb compared to compounds that bind covalently to Hb. Each class of the proposed compound can further be subdivided into three subclasses that differ in their carboxylate arm length. Compounds in each subclass also differ in the number or position of the chloro substituents on the phenoxyalkanoic ring. The synthetic pathways adopted for the preparation of the intermediate and target compounds are outlined in Scheme 1, Scheme 2, Scheme 3, Scheme 4 and Scheme 5 and detailed syntheses are described in the Experimental Section. The compounds were then tested for their effect on sickle RBC morphology and Hb oxygen affinity. 

### 2.2. Test Compounds Showed no Antisickling Effect

**Figure 3:**
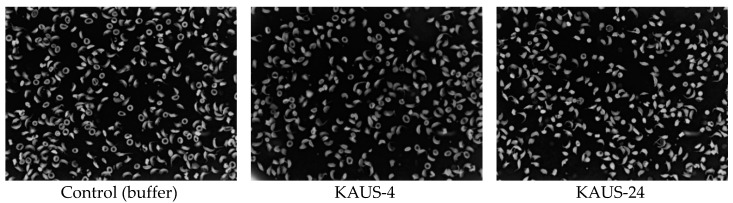
RBC morphological antisickling results of compounds KAUS-4 and KAUS-24. Note that clofibrate and KAUS-23 showed similar prosickling effect as KAUS-24.

The KAUS compounds were expected to bind to the αTrp14 site and prevent sickling of red blood cells through polymer destabilization. We therefore tested selected compounds, including KAUS-4, KAUS-23 and KAUS-24, and the control clofibrate (2 mM) on their effect on sickle RBC morphology as previously reported [18,19,22]. Unexpectedly, the antisickling study showed KAUS-4 to have almost no effect on the RBC morphology, while both KAUS-23 and KAUS-24 actually induced sickling (Figure 3).

Clofibrate acted similarly to KAUS-23 and KAUS-24. It is apparent that our structural modifications failed to have the desired biological effect. Based on this result we speculated that the KAUS compounds instead of binding to the αTrp14 site, are probably binding to the central water cavity of the protein to decrease the protein affinity for oxygen, resulting in the observed biological activities. We therefore undertook both oxygen equilibrium curve and crystallographic studies to understand the biological behavior of the compounds.

### 2.3. Compounds Either Showed no Allosteric Activity or Produced Low-Affinity Hemoglobin

All the KAUS compounds, as well as clofibrate, were studied at 2 mM concentration to determine their effect on Hb oxygen binding property using normal whole blood following published procedures [18,19,22]. The results are shown in Table 1 and Table 2.

Compounds that increase or decrease the oxygen affinity of Hb are expected to shift the OEC to the left or right, respectively, and the degree of shift is reported as a decrease or increase in P_50_ (the oxygen tension at 50% Hb O_2_ saturation). Clofibrate slightly decreased the protein affinity for oxygen (ΔP_50_ of 2 mmHg) consistent with its prosickling activity. The KAUS compounds either showed no significant effect on Hb allosteric activity, as observed with the (imidazole-2-carbonyl)phenoxy-alkanoic acids KAUS 1–8 (Table 1) or marginally decreased the protein affinity for oxygen as observed with the (imidazol-2-yl)phenoxyalkanoic acids KAUS 20–27 (Table 2). Among the three subclasses of the (imidazol-2-yl)phenoxyalkanoic acids (different carboxylate chain lengths), the compounds with chloro substitution at the 2-position (KAUS-22, KAUS-23 and KAUS-24) produced the most low-affinity Hb with ΔP_50_ shift of 2.9, 2.5 and 3.4 mmHg, respectively, with no apparent correlation between the varying carboxylic arm length and the P_50_ shift. It is evident that the KAUS compounds generally did not increase Hb oxygen affinity and thus not expected to prevent hypoxia-induced RBC sickling. It is also apparent that the compounds that produced low-affinity Hb, e.g., KAUS-23 and KAUS-24 are the ones that promoted sickling, while KAUS-4 which showed almost no effect on Hb oxygen binding properties virtually have no effect on RBC morphology. These observations in addition to the antisickling results confirm our suspicion that the compounds do not bind to the αTrp14 site as predicted by our design, but most likely bind to the central water cavity. X-ray crystallographic study was then conducted to determine the actual binding site of these compounds.

### 2.4. Structural Studies Showed KAUS-23 Bound Exclusively to the Central Water Cavity of Hemoglobin

Attempts were made to co-crystallize KAUS-4, KAUS-23, and KAUS-24 with deoxygenated Hb using high-salt conditions as previously published [22,34].

X-ray quality T-state crystals were obtained only for KAUS-23, which crystallized with a tetramer in the asymmetric unit and in space group P2_1_. The crystals are isomorphous with the native deoxygenated Hb crystal (PDB code 2DN2) [35], necessitating the use of 2DN2 as the starting model for the refinement. Structural statistics are summarized in Table 3. The structure has been deposited in the PDB with the ID code 5KDQ. 

**Figure 4:**
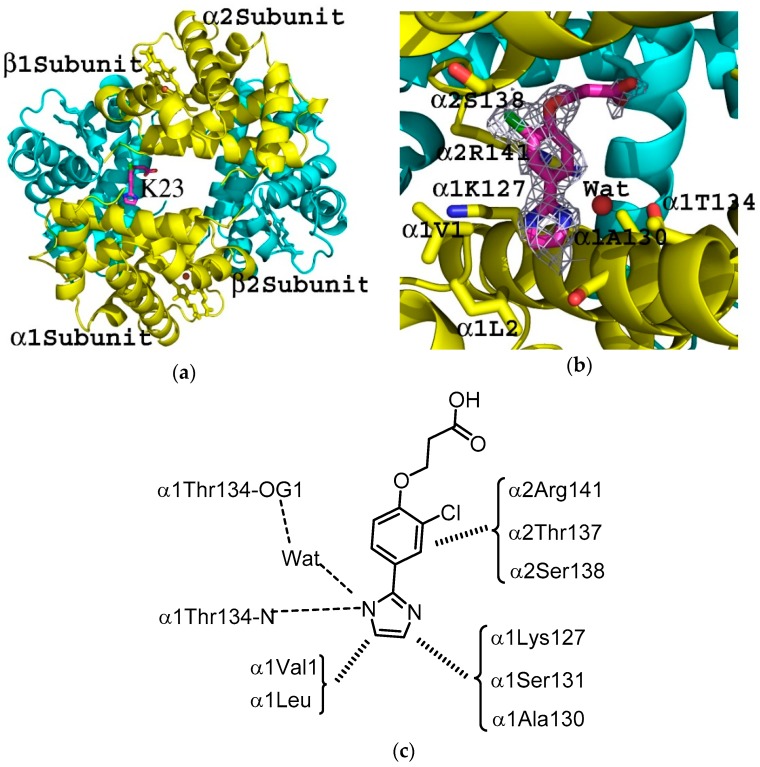
Tetrameric structure of deoxygenated Hb (ribbon) in complex with KAUS-23 (K23; sticks). (**a**) KAUS-23 bound in the central water cavity. Note that only one of the symmetry-related molecules was modelled since the electron density of the second site was too weak and disordered. The α-subunits are colored in yellow and β-subunits in cyan; (**b**) Close-up view of KAUS-23 binding with the final 2Fo-Fc refined electron density map contoured at 0.8σ. For clarity, not all binding site residues shown but described in the text; (**c**) Schematic representation of the interactions between KAUS-23 and the protein at the central water cavity.

As suspected from the OEC and antisickling results, the complex structure showed exclusive binding of KAUS-23 in the central water cavity, close to the α-cleft with no apparent binding at the expected αTrp14 binding site (Figure 4a). We expected two molecules of KAUS-23 to bind, however, only one of the compounds was included in the final structural model as the density at the symmetry-related site was too weak and broken to allow fitting of the second compound. Water molecules were instead modelled into the broken densities (Figure S1).

Although the imidazole and chlorophenoxy moieties show relatively well-defined density, the carboxylate group was characterized by weak and highly disordered electron densities (Figure 4b). The imidazole moiety is located in a hydrophobic pocket formed by α1Val1, α1Leu2, α1Lys127, α1Ser131 and α1Ala130 (Figure 4c). One of the nitrogen atoms of the imidazole forms a water-mediated hydrogen-bond interaction with the side chain of α1Thr134, as well as a direct hydrogen-bond interaction with the main-chain nitrogen atom. Note that all these reactions involve residues from the same α-subunit. The chlorophenoxycarboxylic acid moiety directs further down the central water cavity, where the chlorophenyl group makes inter-subunit hydrophobic interactions with α2Arg141, α2Thr137 and α2Ser138. As noted above, the carboxylate is characterized by very weak density, consistent with the apparent lack of interaction with the protein. It is notable that the KAUS-23 binding site overlaps the chloride ion binding sites at the α-cleft and could potentially inhibit chloride ion binding and influence the Bohr effect [36]. 

Interestingly, when KAUS-23 is compared to clofibrate, the two compounds bind at different sites in the central water cavity. Note that the coordinate for the deoxygenated Hb-clofibrate structure is not available, but clofibrate binding at the central water cavity has been described in published articles at a site surrounded by the αTyr140, αPro95, αVal96, βTrp37, βAsn108 and αLys99 [27]. Bezafibrate, urea derivatives of bezafibrate and RSR13 (Efaproxiral) are potent allosteric effectors that were designed based on clofibrate binding to the central water cavity [2,6,30,31,37,38]. Expectedly, the high-resolutions structures of these compounds in complex with Hb show them overlapping with clofibrate, allowing for better atomic level understanding of how clofibrate binds and stabilizes the T-state Hb. Specifically, these effectors interact with one β-chain and two α-chains that involve αHis103, αLys99, βAsn108, βTyr35, βTrp37 and αVal96; residues that are known to surround clofibrate binding. Notably, these residues are known to shift significantly during the T→R transition and their interactions with the effectors freeze them from moving to the R-state position [1,2]. 

Like clofibrate, bezafibrate, RSR13 and several of their analogs and derivatives, the inter-molecular mediated interaction of KAUS-23 with the protein is expected to stabilize the T-state [1,2], consistent with its observed allosteric activity of decreasing the protein affinity for oxygen. We expect other KAUS compounds to bind in a similar fashion in the central water cavity, although subtle differences in their binding modes may have different stabilization effect on the T-state, explaining the differences in their allosteric properties. This is consistent with several oxygen binding and crystallographic studies that show effectors binding at the same site of Hb yet shift the oxygen equilibrium curve by different degrees [1,2,39]. We are currently working to co-crystallize Hb and the KAUS compounds that exhibit a wide range of activity that are likely to provide additional explanation for their properties.

## 3. Experimental Section

### 3.1. Materials

Normal whole blood was collected from adult donors at the Virginia Commonwealth University (Richmond, VA, USA) after informed consent. Hb was purified from discarded normal blood samples following a published procedure [34]. The use of these human samples is in accordance with regulations of the IRB for Protection of Human Subjects. For the sickling assays, leftover blood samples from patients with homozygous SS were utilized, based on an approved IRB protocol at the Children’s Hospital of Philadelphia (Philadelphia, PA, USA), with informed consent.

### 3.2. Chemistry

Except as otherwise indicated, all reactions were carried out under a nitrogen atmosphere in flame- or oven-dried glassware. Reagents and solvents for chemical synthesis were purchased from Sigma-Aldrich (St. Louis, MO, USA; through Bayouni Imp. Inc., Dist., Jeddah, Saudi Arabia), Alfa Aesr (Haverhill, MA, USA; through Saggaf Co., Dist, Jeddah, Saudi Arabia) or Acros Organics (Geel, Belgium; through Abdullatif H. Abuljadayel Est., Dist, Jeddah, Saudi Arabia) as ACS-reagent grade; and used without further purification. Tetrahydrofuran (THF) was distilled from sodium/benzophenone-ketyl. Reactions were monitored by thin layer chromatography (TLC) with 0.25-mm pre-coated silica gel plates (E. Merck, Billerica, MA, USA). ^1^H-NMR spectra were recorded on an AV-300 NMR spectrometer (Bruker Billerica, MA, USA) equipped with the Top Spin software. Infrared spectra were recorded on a Bruker ATIR spectrometer. Melting points were recorded on a Buchi (Flawil, Switzerland; Bayouni Imp. Inc.) melting point apparatus and were uncorrected. LCMS were run on an Agilent 6130 Series (Santa Clara, CA, USA), single quad instrument. HPLC separation was run on 1200 SERIES HPLC using Chemstation Software (B.02, Agilent). Purities were assessed by HPLC and were confirmed to be >95% for all final compounds. Elemental analyses were performed using Vario, an Elementary apparatus (Shimadzu, Kyoto, Japan), located at the Organic Microanalysis Unit, Cairo University (Giza, Egypt). Column chromatography was performed on silica gel 60 (particle size 0.06–0.20 mm). Jones’ reagent was prepared by adding glacial acetic acid (6 mL) drop wise to an ice cold solution of CrO_3_ (6.7 g) in water (50 mL). The orange solution was stirred for 30 min at the same temperature. Freshly prepared solution was used for oxidation reactions.

### 3.3. General Procedure for the Synthesis of 2,3-Di/monosubstituted-4-[(1-trityl-1H-imidazol-2-yl)-hydroxymethyl]phenols ***3a**–**c*** [40]

To a solution of 1-tritylimidazole (8.12 g, 26.160 mmol) in anhydrous THF (165 mL) was added *n*-BuLi (1.28 M in THF, 20.0 mL, 1.67 g, 13.08 mmol) at −20 °C over a period of 20 min under nitrogen atmosphere. The red solution was allowed to attain room temperature and stirred for 1 h, then cooled to −78 °C. In a separate flask the appropriate aldehyde **1a**–**c** (10.47 mmol) was dissolved in anhydrous THF (4 mL) and added to the red solution dropwise at −78 °C. The reaction mixture was stirred at −78 °C for 1 h and slowly brought to room temperature during which red color tuned to yellow and then to colorless. After complete reaction, saturated NH_4_Cl (250 mL) was added to the reaction mixture at −78 °C. The resulting mixture was extracted with EtOAc (3 × 100 mL); the organic layer was separated, washed with water, saturated NaCl, and dried over anhydrous Na_2_SO_4_. The organic layer was evaporated in vacuo and the residue washed with cold CH_2_Cl_2_.

*2,3-Dichloro-4-[(1-trityl-1H-imidazol-2-yl)hydroxymethyl]phenol* (**3a**): white solid (3.99 g, 76%), m.p. 190.0–191.8 °C. ^1^H-NMR (DMSO-*d*_6_) δ 10.42 (s, 1H, OH), 7.25–7.21 (m, 9H, trityl-H), 7.08 (d, 2H, *J* = 8.7 Hz, Ar-H), 7.00–6.98 (m, 6H, trityl-H), 6.67 (d, 2H, *J* = 4.8 Hz, imidazole-H), 5.25 (d, 1H, *J* = 7.8 Hz, OH), 5.18 (d, 1H, *J* = 7.8 Hz, CH).

*2-Chloro-4-[(1-trityl-1H-imidazol-2-yl)hydroxymethyl]phenol* (**3b**): white solid (2.76 g 93%), m.p. 165.2–166.5 °C. ^1^H-NMR (DMSO-*d*_6_) δ 9.83 (s, 1H, OH), 7.35–7.33 (m, 9H, trityl-H), 7.09–7.07 (m, 6H, trityl-H), 6.96 (s, 1H, Ar-H3), 6.57–6.39 (m, 3H, Ar-H and imidazole-H), 6.37 (d, 1H, *J* = 8.7 Hz, imidazole-H), 5.38 (d, 1H, *J* = 6.6 Hz, OH), 4.82 (d, 1H, *J* = 6.3 Hz, CH).

*3-Chloro-4-[(1-trityl-1H-imidazol-2-yl)hydroxymethyl]phenol* (**3c**): white solid (2.1 g, 70.4%), m.p. 196.0–197.7 °C. ^1^H-NMR (DMSO-*d*_6_) δ 9.59 (s, 1H, OH), 7.24–7.00 (m, 10H, Ar-H and trityl-H), 6.99–6.65 (m, 6H, trityl-H), 6.64 (s, 1H, Ar-H2), 6.49–6.43 (m, 3H, Ar-H and imidazole-H), 5.23 (d, 1H, *J* = 6.6 Hz, OH), 4.90 (d, 1H, *J* = 6.6 Hz, CH).

### 3.4. General Procedure for the Synthesis of Ethyl {2,3-Di/monosubstituted-4-[(1-trityl-1H-imidazol-2-yl)-hydroxymethyl]phenoxy}esters ***5a**–**f*** [41]

To a solution of the appropriate alcohol **3** (7.75 mmol) in anhydrous DMF (45 mL) was added K_2_CO_3_ (3.082 g, 22.300 mmol) at room temperature and stirred for 1 h. To the reaction mixture bromo ethyl esters **4** (10.70 mmol) was added and stirred for 5–6 h at room temperature. Water (450 mL) was added, and the precipitated solid was filtered and dried. The crude products were used as such in the next step without further purification.

*Ethyl {2,3-dichloro-4-[(1-trityl-1H-imidazol-2-yl)hydroxymethyl]phenoxy}acetate* (**5a**): white solid (4.82 g, 94%), m.p. 169.1–171.5 °C. The ^1^H-NMR (DMSO-*d*_6_) δ 7.39–7.10 (9H, m, trityl-H), 6.98–6.81 (m, 7H, trityl-H and Ar-H), 6.80 (d, 1H, *J* = 9 Hz, Ar-H), 5.56 (d, 1H, *J* = 6.9 Hz, OH), 4.80 (d,1H, *J* = 6.9 Hz, CH), 4.75 (s, 2H, OCH_2_), 4.18 (q, 2H, *J* = 6.9 Hz, CH_2_), 1.24 (t, 3H, *J* = 6.9 Hz, CH_3_).

*Ethyl {2-chloro-4-[(1-trityl-1H-imidazol-2-yl)hydroxymethyl]phenoxy}acetate (***5b**): white solid (2.0 g, 88.8%), m.p. 161.5–163.9 °C. ^1^H-NMR (DMSO-*d*_6_) δ 7.39–7.33 (m, 9H, trityl-H)*,* 7.10–7.07 (m, 6H, trityl-H), 6.98 (s, 1H, Ar-H3), 6.66 (d, 1H, *J* = 8.7 Hz, Ar-H6), 6.57–6.49 (m, 3H, Ar-H5 and imidazole-H), 5.55 (s, 1H, OH), 4.87 (s, 1H, CH), 4.75 (s, 1H, OCH_2_), 4.16 (q, 2H, *J* = 7.1 Hz, CH_2_), 1.22 (t, 3H, *J* = 6.9 Hz, CH_3_).

*Ethyl {3-chloro-4-[1-trityl-1H-imidazol-2-yl)hydroxymethyl]phenoxy}acetate (***5c**): white solid (1.5 g, 84.4%), m.p. 189.3–192.3 °C. ^1^H-NMR (DMSO-*d*_6_) δ 7.30–7.22 (m, 10H, Ar-H and trityl-H), 7.08 (s, 1H, Ar-H2), 7.01–6.98 (m, 6H, trityl-H), 6.68–6.63 (m, 3H, Ar-H and imidazole-H), 5.25 (d, 1H, *J* = 6.9 Hz, OH), 5.12 (d, 1H, *J* = 6.9 Hz, CH), 4.75 (s, 2H, OCH_2_), 4.16 (q, 2H, *J* = 7.2 Hz, CH_2_), 1.23 (t, 3H, *J* = 6 Hz, CH_3_). 

*Ethyl 4-{[2,3-Dichloro-4-(1-trityl-1H-imidazol-2-yl)hydroxymethyl]phenoxy}butyrate (***5d**): white solid (3.0 g, 97.7%), m.p. 192.1–193.6 °C. ^1^H-NMR (DMSO-*d*_6_) δ 7.21–7.17 (m, 19H, trityl-H, Ar-H and imidazole-H), 5.8 (s, 1H, OH), 5.33 (t, 2H, *J* = 9 Hz, OCH_2_), 4.07 (m, 3H*,* CH and OCH_2_), 2.5 (2H, under DMSO, CH_2_), 1.98 (t, 2H, *J* = 6.6 Hz, CH_2_), 1.17 (t, 3H, *J* = 6.9 Hz, CH_3_).

*Ethyl 4-{2-chloro-4-[(1-trityl-1H-imidazol-2-yl)hydroxymethyl]phenoxy}butyrate (***5e**): white solid (4.1 g, 66%), m.p. 161.5–164.9 °C.^1^H-NMR (DMSO-*d*_6_) δ_H_ 7.33–7.20 (m, 9H, trityl-H), 7.09–7.08(m, 7H, trityl-H and Ar-H), 6.97 (s, 1H, Ar-H3), 6.73 (d, 1H, *J* = 8.7 Hz, Ar-H6), 6.56–6.54 (m, 2H, imidazole-H), 5.52 (s, 1H, OH), 4.86 (s, 1H, CH), 4.05 (q, 2H, *J* = 6.9 Hz*,* OCH_2_), 3.97 (t, 2H, *J* = 7.2, 6.9 Hz, OCH_2_), 2.45 (2H under DMSO, CH_2_), 1.93 (t, 2H, *J* = 6.6 Hz, CH_2_), 1.17 (t, 3H, *J* = 6. 9 Hz, CH_3_).

*Ethyl 4-{3-Chloro-4-[(1-trityl-1H-imidazol-2-yl)hydroxymethyl]phenoxy}butyrate (***5f**): white solid (3.7 g, 84.95%), m.p. 147.5–148.8 °C. ^1^H-NMR (DMSO-*d*_6_) δ_H_ 7.31–7.21 (m, 10H, trityl-H and ArH), 7.08 (s, 1H, Ar-H2), 7.01–6.99 (m, 6H, trityl-H), 6.66–6.61 (m, 3H, Ar-H and imidazole-H), 5.26 (s, 1H, OH), 5.06 (s, 1H, CH), 4.07 (q, 2H, *J* = 7.2, 6.9 Hz, CH_2_), 3.94(t, 2H, *J* = 6, 5.7 Hz, OCH_2_), 2.46 (under DMSO, CH_2_), 1.94(t, 2H, *J* = 6.6, 6.3 Hz, CH_2_), 1.19 (t, 3H, *J* = 6.9 Hz, CH_3_). 

### 3.5. General Procedure for the Synthesis of Ethyl {[2,3-Di/monosubstituted-4-(1-trityl-1H-imidazol-2-yl)carbonyl]phenoxy}esters ***6a**–**f*** [42]

To a solution of alcohol **5** (7.659 mmol) in acetone (45 mL), Jones reagent (30.6 mL) at 0 °C, was added. The reaction mixture was allowed to attain room temperature and stirred for 7–8 h. Then, water (50 mL) was added to the reaction mixture and extracted with CH_2_Cl_2_ (3 × 100 mL). The organic layer was separated, washed with water, followed by saturated NaCl. After drying over anhydrous Na_2_SO_4_, the organic layer was evaporated in vacuo; the obtained solid was collected and dried. The crude product was used as such in the next step without further purification.

*Ethyl {[2,3-dichloro-4-(1-trityl-1H-imidazol-2-yl)carbonyl]phenoxy}acetate* (**6a**): pale yellow solid (2.8 g, quantitative), m.p. 116.0–120.5 °C. ^1^H-NMR (DMSO-*d*_6_) δ_H_ 7.62 (d, 2H, *J* = 8.7 Hz, Ar-H), 7.30–7.19 (m, 15H, trityl-H), 6.45 (d, 2H, *J* = 1. 2 Hz, imidazole-H), 5.07 (s, 2H, OCH_2_), 4.19 (q, 2H, *J* = 7.2, 6.9 Hz, CH_2_), 1.23 (t, 3H, *J* = 7.2, 6.9 Hz, CH_3_). 

*Ethyl {[2-Chloro-4-(1-trityl-1H-imidazol-2-yl)carbonyl]phenoxy}acetate (***6b**): white solid (1.1 g, quantitative), m.p. 160.3–161.6 °C. ^1^H-NMR (DMSO-*d*_6_) δ_H_ 7.08–7.53 (m, 19H, trityl-H, Ar-H and imidazole-H), 6.46 (s, 1H, Ar-H3), 5.07 (s, 2H, OCH_2_), 4.20 (q, 2H, *J* = 7.2, 6.9 Hz, CH_2_), 1.23 (t, 3H, *J* = 7.2, 6.9 Hz, CH_3_).

*Ethyl {[3-Chloro-4-(1H-imidazol-2-yl)carbonyl]phenoxy}acetate (***6c**): white solid (0.9 g, quantitative), m.p. 171.5–172.0 °C. ^1^H-NMR (DMSO-*d*_6_) δ_H_ 13.56 (s, 1H, NH), 7.74 (d, 1H, *J* = 6.9 Hz, Ar-H5), 7.54 (d, 1H, *J* = 1.2 Hz, Ar-H2), 7.22–7.01 (m, 3H, imidazole-H and Ar-H6), 4.95 (s, 2H, OCH_2_), 4.20 (q, 2H, *J* = 7.2, 6.9 Hz, CH_2_), 1.24 (t, 3H, *J* = 7.2, 6.9 Hz, CH_3_). 

*Ethyl 4-{[2,3-Dichloro-4-(1-trityl-1H-imidazol-2-yl)carbonyl]phenoxy}butyrate* (**6d**): pale yellow solid (1.8 g, quantitative). The crude product was used as such in the next step without further purification. ^1^H-NMR (DMSO-*d*_6_) δ_H_ 7.33–7.19 (m, 19H, trityl-H, Ar-H and imidazole-H), 4.22 (t, 2H, *J* = 6.3, 6 Hz, OCH_2_), 4.07 (q, 2H, *J* = 7.2, 3.9 Hz, CH_2_), 3.55 (under DMSO, CH_2_), 3.05 (m, 2H,CH_2_), 1.18 (t, 3H**,** *J* = 7.2 Hz, CH_3_).

*Ethyl 4-{[2-Chloro-4-(1-trityl-1H-imidazol-2-yl)carbonyl]phenoxy}butyrate* (**6e**): white solid (3.7 g, quantitative), m.p. 169.1–171.5 °C. ^1^H-NMR (DMSO-*d*_6_) δ_H_ 7.30–7.19 (m, 19H, trityl-H, Ar-H and imidazole-H), 6.46 (s, 1H, Ar-H3), 4.22 (t, 2H, *J* = 6.3, 6 Hz, OCH_2_), 4.07 (q, 2H, *J* = 7.2, 6.9 Hz, OCH_2_), 3.55 (under DMSO, CH_2_), 2.07-2.03 (m, 2H,CH_2_), 1.18 (t, 3H**,** *J* = 7.2, 6.9 Hz, CH_3_).

*Ethyl 4-{[3-Chloro-4-(1H-imidazol-2-yl)carbonyl]phenoxy}butyrate* (**6f**): white solid (1.8 g, 89.74%), m.p. 112.1–114.0 °C. ^1^H-NMR (DMSO-*d*_6_) δ_H_ 13.52 (s, 1H, NH), 7.75 (d, 1H, *J* = 8.4 Hz, Ar-H5), 7.53 (s, 1H, Ar-H2), 7.22–6.99 (m, 3H, ArH6 and imidazole-H), 4.11–4.10 (m, 4H, two OCH_2_), 2.51 (under DMSO, CH_2_), 1.99 (t, 2H, *J* = 6.6 Hz, CH_2_), 1.19 (t, 3H, *J* = 6.9 Hz, CH_3_).

### 3.6. General Procedure for the Synthesis of {[2,3-Di/monosubstituted-4-(1H-imidazol-2yl)carbonyl]-phenoxy}acetic/butyric acids (***7a**–**f***) [43]

To solution of **6** (8.159 mmol) in aqueous THF (12 mL/ water 6 mL) was added LiOH·H_2_O (0.687 g, 16.37 mmol) at room temperature. The reaction mixture was stirred for 1 h during which the solution turned to green. The reaction was monitored with TLC. After complete reaction, the solvent was evaporated in vacuo. The crude material was then dissolved in minimum amount of water. The precipitated solid was filtered and the pH of the filtrate was adjusted to 6–7 using aqueous 1.5 N HCl, and the obtained solid product was collected by filtration and dried. The crude material was purified by column chromatography over silica gel (230–400 mesh) using 40 to 50% ethyl acetate in hexane as eluent to afford the desired final products **7a**–**i**.

*{[2,3-Dichloro-4-(1H-imidazol-2-yl)carbonyl]phenoxy}acetic acid*, (**7a**): white solid (1.22 g, 51%), m.p. 240.2–243 °C. IR (KBr, ν_max_ cm^−1^): 3527 (OH), 3200 (NH), 1662 (CO), 1269, 1077 (C-O-C); ^1^H-NMR (DMSO-*d*_6_) δ_H_ 13.64 (s, 1H, NH), 7.64–7.57 (m, 2H, Ar-H), 7.22–7.13 (m, 2H, imidazole-H), 4.95 (s, 2H, OCH_2_); LC/MS (ESI) *m*/*z* 315 (M + 1), 316 (M + 2), 317 (M + 3), 318 (M + 4).

*{[2-Chloro-4-(1H-imidazol-2-yl)carbonyl]phenoxy}acetic acid* (**7b**): pale yellow solid (1.04 g, 81.7%), m.p. 303–306 °C. ^1^H-NMR (DMSO-*d*_6_) δ_H_ 13.42 (brs, 1H, NH), 8.76 (d, 1H, *J* = 1.8 Hz, Ar-H3), 8.39 (t, 1H, *J* = 9, 1.8 Hz, Ar-H5), 7.38 (s, 2H, imidazole-H), 6.97 (d, 1H, *J* = 9 Hz, Ar-H6), 4.38 (s, 2H, OCH_2_); LC/MS (ESI) *m*/*z* 280.1 (M + ), 282.1 (M + 2).

*{3-Chloro-4-[(1H-imidazol-2-yl)carbonyl]phenoxy}acetic acid* (**7c**): white solid (0.64 g, 64.0%), m.p. 230.8–233.9 °C. IR (KBr, ν_max_ cm^−1^): 3497 (NH), 3306 (OH), 1647 (CO), 1269, 1085 (C-O-C); ^1^H-NMR (DMSO-*d*_6_) δ_H_ 13.56 (s, 1H, NH), 7.72 (d, 1H, *J* = 8.7 Hz, Ar-H5), 7.51 (s, 1H, imidazole-H), 7.20 (s, 1H, imidazole-H), 6.96 (d, 1H, *J* = 2.1 Hz, Ar-H2), 6.88 (t, 1H, *J* = 6.3, 2.4 Hz Ar-H6), 4.40 (s, 2H, OCH_2_); LC/MS (ESI) *m*/*z* 280.1 (M + )^−^, 282.1 (M + 2)^−^.

*4-{2,3-Dichloro-4-[(1H-imidazol-2-yl)carbonyl]phenoxy}butyric acid* (**7d**): white solid (1.15 g, 69%), m.p. 167.5–169.4 °C. IR (KBr, ν_max_ cm^−1^): 3527 (NH), 3247 (OH), 1643 (CO), 1274, 1041 (C-O-C). ^1^H-NMR (DMSO-*d*_6_) δ_H_ 13.61 (s, 1H, NH), 7.64 (d, 1H, *J* = 8.4 Hz, Ar-H5), 7.36 (s, 2H, imidazole-H), 7.29 (d, 1H, *J* = 8.7 Hz, Ar-H6), 4.19 (t, 2H, *J* = 6.6 Hz, OCH_2_), 2.13 (t, 2H, *J* = 6.6 Hz, OCH_2_), 1.94 (q, 2H, *J* = 6.6, 6.3 Hz, CH_2_). LC/MS (ESI) *m*/*z* 341 (M − 1)^−^, 343 (M + 1)^−^, 344 (M + 2)^−^, 346 (M + 4)^−^.

*4-{2-Chloro-4-[(1H-imidazol-2-yl)carbonyl]phenoxy}butyric acid* (**7e**): white solid (2.4 g, 71%), m.p. 169.1–171.5 °C. ^1^H-NMR (DMSO-*d*_6_) δ_H_ 13.52 (brs, 1H, NH), 8.73 (s, 1H, Ar-H3), 8.52 (d, 1H, *J* = 7.8 Hz, Ar-H5), 7.41–7.32 (m, 3H, Ar-H6 and imidazole-H), 4.20 (t, 2H, *J* = 6 Hz, OCH_2_), 2.3 (t, 2H, *J* = 6 Hz, CH_2_), 1.99–1.97(m, 2H, CH_2_). LC/MS (ESI) *m*/*z* 307.1 (M − 1)**^−^**, 308 (M + )**^−^**, 309.1 (M + 1)**^−^**, 310 (M + 2)**^−^**, 312 (M + 4)**^−^**.

*4-{3-Chloro-4-[(1H-imidazole-2-yl)carbonyl]phenoxy}butyric acid* (**7f**): white solid (0.8 g, 48.5%), m.p. 188.4–191.1 °C. ^1^H-NMR (DMSO-*d*_6_) δ_H_ 13.53 (s, 1H, NH), 12.19 (s,1H,OH), 7.76 (d, 1H, *J* = 8.7 Hz, Ar-H5), 7.52 (s, 1H, Ar-H2), 7.23 (s, 1H, imidazole-H), 7.13(s, 1H, imidazole-H), 7.02 (d, 1H, *J* = 8.4 Hz, Ar-H6), 4.10 (t, 2H, *J* = 6.3, 6 Hz, OCH_2_), 2.40 (2H, under DMSO, CH_2_), 1.97 (m, 2H, CH_2_); LC/MS (ESI) *m*/*z* 307 (M − 1)^−^, 309.1 (M + 1)^−^, 310.1 (M + 2)^−^, 312 (M + 4)^−^.

### 3.7. General Procedure for the Synthesis of 3-{2,3-Di/monochloro-4-[(1-trityl-1H-imidazol-2-yl)hydroxy-methyl]phenoxy}propanols ***9a**–**c*** [41]

To a solution of the appropriate alcohol **3a**–**c** (12.76 mmol) in anhydrous DMF (65 mL) was added K_2_CO_3_ (4.4 g, 31.84 mmol) at room temperature and stirred for 1 h. To the reaction mixture, 3-bromopropanol (2.12 g, 1.4 mL, 15.31 mmol) was added and stirred for 16 h at room temperature. The solid formed after addition of water (650 mL) was filtered, treated with diethyl ether and dried in vacuo.

*3-{2,3-Dichloro-4-[(1-trityl-1H-imidazol-2-yl)hydroxymethyl]phenoxy}propanol* (**9a**): white solid (6.34 g, 88.78%), m.p. 174.7–177.7 °C. ^1^H-NMR (DMSO-*d*_6_) δ_H_ 7.25–7.10 (m, 11H, trityl-H and Ar-H), 7.0–6.9 (m, 7H, trityl-H and imidazole-H), 6.87 (d, 1*H, J* = 8.4 Hz, imidazole-H), 5.29 (s, 2H, OH and CH), 4.6 (brs, 1H, OH), 4.09 (t, 2H, *J* = 6, 6.3 Hz, OCH_2_), 3.57 (t, 2H, *J* = 6, 6.3 Hz, OCH_2_), 1.83 (quint, 2H, *J* = 6.3 Hz, CH_2_).

*3-{2-Chloro-4-[(1-trityl-1H-imidazol-2-yl)hydroxymethyl]phenoxy}propanol* (**9b**): white solid (3.9 g, 88.94%), m.p. 155.9–157.5 °C. ^1^H-NMR (DMSO-*d*_6_) δ_H_ 7.34–7.33 (m, 9H, trityl-H), 7.10–7.08 (m, 6H, trityl-H), 6.97 (s, 1H, Ar-H3), 6.74 (d, 1H, *J* = 8.4 Hz, Ar-H6), 6.57–6.51 (m, 3H, ArH and imidazole-H), 5.57 (brs, 1H, OH), 4.86 (s, 1H, CH), 4.59 (brs, 1H, OH), 4.01 (t, 2H, *J* = 6, 6.3 Hz, OCH_2_), 3.54 (t, 2H, *J* = 6, 6.3 Hz, OCH_2_), 1.83 (quint, 2H, *J* = 6.3 Hz, CH_2_).

*3-{3-Chloro-4-[(1-trityl-1H-imidazol-2-yl)hydroxymethyl]phenoxy}propanol* (**9c**): white solid (4.5 g, 87.0%), m.p. 182.3–183.9 °C. ^1^H-NMR (DMSO-*d*_6_) δ_H_ 7.31–7.28 (m, 10H, Ar-H and trityl-H), 7.22 (s, 1H, Ar-H2), 7.08–7.00 (m, 6H, trityl-H), 6.66–6.60 (m, 3H, ArH and imidazole-H), 5.26 (s, 1H, CH), 5.04 (brs, 1H, OH), 4.56 (brs, 1H, OH), 3.97 (t, 2H, *J* = 6, 5.4 Hz, OCH_2_), 3. 49 (t, 2H, *J* = 6, 6.3 Hz, OCH_2_), 1.81 (quint, 2H, *J* = 6, 5.7 Hz, CH_2_).

### 3.8. General Procedure for the Synthesis of 3-{2,3-Di/monochloro-4-[(1H-imidazol-2-yl)carbonyl]phenoxy}-propionic Acids ***7g**–**i*** [42]

To a solution of alcohol **9a**–**c** (10.72 mmol) in acetone (60 mL), Jones reagent (25 mL) was added at 0 °C. The reaction mixture was allowed to attain room temperature and stirred for 48 h and filtered to remove insoluble impurities. The filtrate was extracted with diethyl ether (6 × 50 mL). The organic layer was separated, dried over anhydrous Na_2_SO_4_ and evaporated in vacuo to get a pale yellow solid. The product was dissolved in 5% NaHCO_3_ solution (minimum amount) and washed with diethyl ether (2 × 50 mL) followed by ethyl acetate (2 × 50 mL). The pH of the aqueous layer was carefully adjusted to 6 using 1.5 N aqueous HCl. The solid product formed was filtered, washed with diethyl ether and dried. 

*3-{2,3-dichloro-4-[(1H-imidazol-2-yl)carbonyl]phenoxy}propionic acid,* **7g**: white solid (0.45 g, 12.75%), m.p. 207 °C (decomposed). IR (KBr, ν_max_ cm^−1^): 3497(NH), 3306 (OH), 1647(CO), 1600, 1409 (CO_2_^−^), 1270, 1035 (C-O-C). ^1^H-NMR (DMSO-*d*_6_) δ 13.64 (s, 1H, NH), 7.67 (d, 1H, *J* = 8.7 Hz, Ar-H5), 7.40 (s, 2H, imidazole-H), 7.28 (d, 1H, *J* = 8.7 Hz, Ar-H6), 4.36 (t, 2H, *J* = 6 Hz, OCH_2_), 2.68 (2H, *J* = 6.3, 5.7 Hz, CH_2_).

*3-{2-Chloro-4-[(1H-imidazol-2-yl)carbonyl]phenoxy}propionic acid* (**7h**): white solid (1.0 g, 24.08%), m.p. 195.0–195.4 °C. IR (KBr, ν_max_ cm^−1^): 3294 (NH), 3000–2800 (OH), 1703 (CO), 1613(CO), 1583 (C=N), 1254, 1037 (C-O-C). ^1^H-NMR (DMSO-*d*_6_) δ_H_ 13.47 (s, 1H, NH), 12.49(s, 1H, OH), 8.72 (d, 1H, *J* = 1.8 Hz, Ar-H3), 8.53 (t, 1H, *J* = 6.6, 2.1 Hz, Ar-H5), 7.52 (s, 1H, imidazole-H), 7.35(d, 1H, *J* = 8.7 Hz, Ar-H6), 7.31 (s, 1H, imidazole-H), 4.38 (t, 2H, *J* = 6, 5.7 Hz, OCH_2_), 2.78 (t, 2H, *J* = 6, 5.7 Hz, CH_2_). LC/MS (ESI) *m*/*z* 293 (M − 1), 295 (M + 1), 296 (M + 2).

*3-{3-Chloro-4-(1H-imidazol-2-yl)carbonyl]phenoxy}propionic acid* (**7i**): white solid (0.536 g, 21.22%), m.p. 193.0–194.2 °C. IR (KBr, ν_max_ cm^−1^): 3301 (NH), 3100–2600 (OH), 1717 (CO), 1645 (CO), 1598 (C=N), 1294, 1033 (C-O-C). ^1^H-NMR (DMSO-*d*_6_) δ_H_ 13.53 (s, 1H, NH), 12.43 (s, 1H, OH), 7.75 (d, 1H, *J* = 8.7 Hz, Ar-H5), 7.53 (s, 1H, imidazole-H), 7.22 (s, 1H, imidazole-H) 7.14 (d, 1H, *J* = 2.1 Hz, Ar-H2), 7.02 (dd, 1H, *J* = 6.3, 2.4 Hz, Ar-H6), 4.28 (t, 2H, J = 6, 5.7 Hz, OCH_2_), 2.73 (t, 2H, *J* = 6, 5.7 Hz, CH_2_). LC/MS (ESI) *m*/*z* 293 (M − 1), 294 (M + ), 295 (M + 1), 296 (M + 2).

### 3.9. 3-Chloro-4-(1H-imidazol-2-yl)phenol (***11***) [30]

Glyoxal **10** (40% in H_2_O, 14 mL, 0.095 mol) was placed in water (20 mL), then added to a cooled solution (about 5 °C) of 2-chloro-4-hydroxybenzaldehyde **1c** (5 g, 0.031 mmol) in methanol (10 mL) to afford a white turbid solution. Ammonium hydroxide (25% in H_2_O, 67 mL, 0.478 mmol) was added drop wise over a period of 1 h at 0–5 °C and the reaction mixture was stirred for 3 h at 0–5 °C. The mixture was allowed to warm to room temperature and stirred further for 24 h. The solution was concentrated in vacuo and the crude was purified by column chromatography using mixture of DCM/MeOH (95:5) as an eluent to obtain **11** as a beige solid (6.4 g, quantitative), m.p. 155–158 °C. ^1^H-NMR (300 MHz, DMSO-*d*_6_) δ_H_ 12.07 (brs, 1H, NH), 10.20 (brs, 1H, OH), 7.52–7.63 (m, 1H, Ar-H6), 7.10 (s, 1H, Ar-H2), 6.91 (s, 2H, imidazole-H), 6.83 (d, *J* = 8.50 Hz*,* 1H, Ar-H5).

### 3.10. Ethyl 2-[3-Chloro-4-(1H-imidazol-2-yl)phenoxy]acetate (***12***) [42]

To a cold (5–10 °C) solution of **11** (9.0 g, 0.049 mol) and K_2_CO_3_ (1 g, 0.070 mol), KI (0.37 g, 0.005 mol) in DMF (90 mL), was added ethyl bromoacetate (7 g, 0.049 mol) while stirring. The stirring was continued for 1 h at room temperature. Then the reaction was treated with water (150 mL) and extracted in DCM (2 × 100 mL). The combined organic layer was washed with water and brine, dried with anhydrous Na_2_SO_4_, and evaporated under vacuum to give the crude product. Purification was carried out using flash chromatography (hexane–EtOAC 3:7) to furnish a white solid of **12** (1.4 g, 11%), m.p. 131–133 °C. ^1^H-NMR (300 MHz, DMSO-*d*_6_) δ_H_ ppm 12.29 (brs, 1H, NH) 7.67 (d, *J* = 8.78 Hz, 1H, Ar-H5), 7.07–7.21 (m, 3H, Ar-H2 and imidazole-H), 7.03 (dd, *J* = 8.69, 2.55 Hz, 1H, Ar-H6), 4.90 (s, 2H, OCH_2_), 4.19 (q, *J* = 7.08 Hz, 2H, CH_2_), 1.22 (t, *J* = 7.08 Hz, 3H, CH_3_).

### 3.11. 2-[3-Chloro-4-(1H-imidazol-2-yl)phenoxy]acetic acid (***13***) [43]

To a solution of **12** (1.4 g, 0.005 mol) in THF (5.6 mL) and water (2.8 mL) was added LiOH·H_2_O (0.36 g, 0.015 mol) at room temperature and stirred for 30 min until consumption of the starting material. The reaction was treated with 1.5N HCl until the pH of the reaction mixture became 6.5. The precipitated product was filtered and dried under vacuum as an off-white solid, (1.15 g, 92%), m.p. 313–316 °C.^1^H-NMR (300 MHz, DMSO-*d*_6_) δ_H_ ppm 14.75 (brs,1H, NH), 7.81 (s, 2H, imidazole-H), 7.71 (d, *J* =8.69 Hz, 1H, Ar-H5), 7.34 (d, *J* = 2.46 Hz, 1H, Ar-H2), 7.18 (dd, *J* = 8.69, 2.46 Hz, 1H, Ar-H6), 4.88 (s, 2H, OCH_2_); LC-MS (ESI) *m*/*z* 251.9 (M + )^−^, 253.9 (M + 2)^−^; Anal. Calcd for (C_11_H_9_ClN_2_O_3_): C, 52.29; H, 3.59; Cl, 14.03; N, 11.09; Found: C, 52.15; H, 3.24; Cl, 14.40; N, 11.12.

### 3.12. General Procedure for the Synthesis of Ethyl [(2,3-Di/monochloro-4-formyl)phenoxy]acetates ***14a**,**b*** [41]

To a cold mixture of 4-hydroxybenzaldehydes **1a**,**b** (0.01 mol) and K_2_CO_3_ (0.015 mol) in DMF (20 mL), was added ethyl bromoacetate (0.012 mol) at 5–10 °C. The reaction mixture was stirred for 1hr then treated with water (20 mL) and extracted in DCM (2 × 25 mL). The combined organic extract was washed with water and brine then dried over anhydrous MgSO_4_. After evaporation in vacuo, the residue was treated with hexane to give the esters **14a**,**b**.

*Ethyl [(2,3-dichloro-4-formyl)phenoxy]acetate* (**14a**): pale brown solid (2.4 g, 82%), m.p. 134–138 °C. ^1^H-NMR (300 MHz, CDCl_3_) δ_H_ ppm 10.39 (s, 1H, CHO); 7.87 (d, *J* = 8.78 Hz*,* 1H, Ar-H), 6.84 (d, *J* = 8.78 Hz, 1H, Ar-H), 4.83 (s, 2H, OCH_2_), 4.31 (q, *J* = 7.14 Hz, 2H, CH_2_), 1.32 (t, *J* = 7.13 Hz, 3H, CH_3_).

*Ethyl [(2-chloro-4-formyl)phenoxy]acetate* (**14b**): clear liquid (4.3 g, 94% yield); ^1^H-NMR (300 MHz, DMSO-*d*_6_) δ_H_ ppm 9.88 (s, 1H, CHO), 7.93–8.05 (m, 1H, Ar-H3), 7.86 (d, *J* = 8.50 Hz, 1H, Ar-H5), 7.30 (d, *J* = 8.50 Hz, 1H, Ar-H6), 5.09 (s, 2H, CH_2_), 4.25 (q, *J =* 7.04 Hz, 2H), 1.23 (t, *J* = 7.06 Hz, 3H, CH_3_).

### 3.13. General Procedure for the Synthesis of Ethyl 4-[(Di/monochloro-4-formyl)phenoxy]butanoates ***14c**–**e*** [41]

To a solution of **1a**–**c** (0.018 mol) in *N*-methylpyrrolidone (NMP, 30 mL) was added K_2_CO_3_ (0.0275 mol) and KI (1.8 mmol) at room temperature. After stirring for 30 min, ethyl 4-bromobutyrate (3.5 g, 0.0183 mol) was added, and the reaction was continued for 7 h at 60 °C. The reaction mixture was diluted with water (50 mL) and extracted with DCM (3 × 75 mL). The combined organic extract was washed with water then brine and dried over anhydrous Na_2_SO_4_. The solvent was removed under reduced pressure, and the crude product was purified by column chromatography (6% EtOAc in hexane) to give the products **14c**–**e**.

*Ethyl 4-[(2,3-dichloro-4-formyl)phenoxy]butanoate* (**14c**): white solid (4 g, 72%), m.p. 163–165 °C. ^1^H-NMR (300 MHz, DMSO-*d*_6_) δ_H_ ppm 10.19 (s, 1H, CHO), 7.84 (d, *J* = 8.88 Hz, 1H, Ar-H5), 7.32 (d, *J* = 8.78 Hz, 1H, Ar-H6), 4.25 (t, *J* = 6.23 Hz, 2H, CH_2_), 4.07 (q, *J* = 7.08 Hz, 2H, CH_2_), 2.51 (t, 2H, Under DMSO), 2.04 (quin, *J* = 6.77 Hz, 2H, CH_2_), 1.14 (q, *J* = 6.31, 3H, CH_3_).

*Ethyl 4-[(2-chloro-4-formyl)phenoxy]butanoate* (**14d**): clear liquid (7.1 g, 82%); ^1^H-NMR (300 MHz, DMSO-*d*_6_) δ_H_ ppm 9.86 (s, 1H, CHO), 7.94 (s, 1H, Ar-H3), 7.88 (d, *J =* 8.50 Hz, 1H, Ar-H6), 7.35 (d, *J =* 8.50 Hz*,* 1H, Ar-H5), 4.22 (t, *J* = 6.23 Hz, 2H, CH_2_), 4.07 (q, *J* = 7.08 Hz, 2H, CH_2_), 2.39 (2H, under DMSO), 1.96–2.12 (m, 2H, CH_2_), 1.18 (t, *J* = 7.13 Hz, 3H, CH_3_).

*Ethyl 4-[(3-chloro-4-formyl)phenoxy]butanoate* (**14e**): white solid, (5 g, 82%), m.p. 118–121 °C. ^1^H-NMR (300 MHz, DMSO-*d*_6_) δ_H_ 10.19 (s, 1H, CHO), 7.83 (d, *J* = 8.78 Hz, 1H, Ar-H5), 7.18 (d, *J* = 1.70 Hz, 1H, Ar-H3), 7.08 (dd, *J* = 8.78, 2.27 Hz, 1H, Ar-H6), 4.10–4.20 (m, 2H, CH_2_), 4.02–4.10 (m, 2H, CH_2_), 2.41–2.49 (2H, under DMSO, CH_2_), 1.99 (t, *J* = 6.80 Hz, 2H, CH_2_), 1.18 (t, *J* = 7.13 Hz, 3H, CH_3_) 

### 3.14. General Procedure for the Synthesis of Ethyl [Di/monochloro-4-(4,5-dihydro-1H-imidazol-2-yl)phenoxy]-acetate/butanoates ***15a**–**e*** [44]

To a stirred solution of ethyl esters **14a**–**e** (8.6 mmol) in chloroform (25 mL), was added ethylenediamine at 0 to 5 °C. After stirring for 30 min at room temperature, N-bromosuccinimide was added at 0 to 5 °C over 20 min. The reaction mixture was slowly warmed to room temperature and stirred for 16 h. NaHCO_3_ 10% solution (25 mL) was added to quench the reaction, followed by extraction with chloroform (3 × 50 mL). The combined organic extract was concentrated to afford crude product **15a**–**e**.

*Ethyl [2,3-dichloro-4-(4,5-dihydro-1H-imidazol-2-yl)phenoxy]acetate* (**15a**): pale yellow solid (2.5 g, 91%), m.p. 132–134 °C. ^1^H-NMR (300 MHz, DMSO-*d*_6_) δ_H_ 7.45 (d, *J* = 8.69 Hz,1H, Ar-H5), 7.12 (d, *J* = 8.78 Hz, 1H, Ar-H6), 6.79 (brs, 1H, NH), 5.03 (s, 2H, OCH_2_), 4.18 (q, *J* = 7.08 Hz, 2H, CH_2_), 3.59 (brs, 4H, imidazoline-H), 1.22 (t, *J* = 7.03 Hz, 3H, CH_3_).

*Ethyl [2-chloro-4-(4,5-dihydro-1H-imidazol-2-yl)phenoxy]acetate* (**15b**): greyish white solid (3.8 g, 66%), m.p. 124–126 °C. ^1^H-NMR (300 MHz, DMSO-*d*_6_) δ_H_ 7.89 (s, 1H, NH), 7.73 (d, *J* = 8.69 Hz, 1H, Ar-H6), 7.11 (brs, 1H, Ar-H3), 7.12 (d, *J* = 8.69 Hz, 1H, Ar-H5), 4.98 (s, 2H, OCH_2_) 4.18 (q, *J* = 7.02 Hz, 2H, CH_2_), 3.60 (s, 4H, imidazoline-H) 1.22 (t, *J* = 7.08 Hz, 3H, CH_3_).

*Ethyl 4-[2,3-dichloro-4-(4,5-dihydro-1H-imidazol-2-yl)phenoxy]butanoate* (**15c**): pale greyish white solid (4 g, 89%); ^1^H-NMR (300 MHz, DMSO-*d*_6_) δ_H_ 7.48 (d, *J* = 8.59 Hz, 1H, Ar-H6), 7.19 (d, *J* = 8.88 Hz, 1H, Ar-H5) 4.13–4.25 (m, 2H, CH_2_) 4.07 (q, *J* = 6.89 Hz, 2H, CH_2_), 3.60 (m, 4H, imidazoline-H), 2.51 (t, 2H, under DMSO), 1.94–2.10 (m, 2H, CH_2_), 1.18 (t, *J =* 6.76 Hz, 3H, CH_3_).

*Ethyl 4-[2-chloro-4-(4,5-dihydro-1H-imidazol-2-yl)phenoxy]butanoate* (**15d**): T pale greyish white solid (6.2 g, 76%), m.p. 138–140 °C.^1^H-NMR (300 MHz, DMSO-*d*_6_) δ_H_ 7.88 (d, *J* = 1.51 Hz, 1H, Ar-H3), 7.77 (dd, *J* = 8.64, 1.65 Hz, 1H, Ar-H5), 7.22(d, *J* = 8.59 Hz, 1H, Ar-H6), 4.14 (t, *J* = 6.98 Hz, 2H, CH_2_), 4.05 (q, *J* = 7.12 Hz, 2H,CH_2_), 3.62 (m, 4H, imidazoline-H), 2.47 (under DMSO, 2H, CH_2_), 2.01 (m, 2H, CH_2_), 1.18 (t, *J* = 7.08 Hz,3H, CH_3_). 

*Ethyl 4-[3-chloro-4-(4,5-dihydro-1H-imidazol-2-yl)phenoxy]butanoate* (**15e**): pale yellow solid (5 g, 87%), m.p. 137–139 °C. ^1^H-NMR (300 MHz, DMSO-*d*_6_) δ_H_ 7.52 (d, *J* = 8.69 Hz, 1H, Ar-H5), 7.07 (d, *J* = 2.27 Hz, 1H, Ar-H2), 6.95 (dd, *J* = 8.64, 2.31 Hz, 1H, Ar-H6), 3.99–4.10 (m, 4H, imidazoline-H), 3.60 (m, 4H, 2CH_2_), 2.45 (t, *J* = 7.32 Hz, 2H, CH_2_), 1.97 (t, *J* = 6.80 Hz, 2H, CH_2_),1.18 (t, *J* = 7.08 Hz, 3H, CH_3_).

### 3.15. General Procedure for the Synthesis of Ethyl [2,3-Dichloro-4-(1H-imidazol-2-yl)phenoxy]acetate/butanoates ***16a**–**e*** [44]

To a solution of **15a**–**e** (7.8 mmol) in DMSO (25 mL) was added K_2_CO_3_ (8.6 mmol) and diacetoxyiodobenzene (8.6 mmol) at room temperature. The reaction mixture was stirred for 60 min in a dark place, and then water was added followed by extraction with DCM (3 × 50 mL). The organic layer was dried over Na_2_SO_4_ and concentrated under reduced pressure. The residue was subjected to flash column chromatography (EtOAc 2:hexane 1) to afford the products **16a**–**e**.

*Ethyl [2,3-dichloro-4-(1H-imidazol-2-yl)phenoxy]acetate* (**16a**): off-white solid (1.1g, 44%), m.p. 193–194 °C. ^1^H-NMR (300 MHz, DMSO-*d*_6_) δ_H_ 12.31 (brs, 1H, NH), 7.62 (d, *J* = 8.78 Hz, 1H, Ar-H6), 7.06–7.26 (m, 3H, Ar-H and imidazole-H), 5.04 (s, 2H, OCH_2_), 4.19 (q, *J* = 7.05 Hz, 2H, CH_2_), 1.22 (t, *J* = 7.08 Hz, 3H, CH_3_).

*Ethyl [2-chloro-4-(1H-imidazol-2-yl)phenoxy]acetate* (**16b**): greyish white solid (1.5 g, 43%), m.p. 164–166 °C. ^1^H-NMR (300 MHz, DMSO-*d*_6_) δ_H_ 12.47 (brs, 1H, NH), 8.00 (d, *J* = 2.08 Hz, 1H, Ar-H3), 7.82 (dd, *J* = 8.59, 2.17 Hz, 1H, Ar-H5), 4.96 (s, 2H, OCH_2_), 7.15 (m, 3H, Ar-H and imidazole-H), 4.19 (q, *J* = 7.08 Hz, 2H, CH_2_) 1.22 (t, *J* = 7.13 Hz, 3H, CH_3_).

*Ethyl 4-[2,3-dichloro-4-(1H-imidazol-2-yl)phenoxy]butanoate* (**16c**): off-white solid (2.3 g, 55.8%), m.p. 226–227 °C. ^1^H-NMR (300 MHz, CDCl_3_) δ_H_ 10.19 (brs, 1H, NH), 8.14 (d, *J* = 8.88 Hz, 1H, Ar-H6), 7.21 (s, 2H, imidazole-H), 6.98 (d, *J* = 8.97 Hz, 1H, Ar-H5), 4.00–4.34 (m, 4H, 2CH_2_), 2.61 (t, *J* = 7.18 Hz, 2H, CH_2_), 2.21 (quin, *J* = 6.61 Hz, 2H, CH_2_), 1.16–1.44 (m, 3H, CH_3_).

*Ethyl 4-[2-chloro-4-(1H-imidazol-2-yl)phenoxy]butanoate* (**16d**): greyish white solid (3.4 g, 57%), m.p. 179–182 °C. ^1^H-NMR (300 MHz, DMSO-*d*_6_) δ_H_ ppm 12.44 (brs, 1H, NH), 7.98 (d, *J* = 1.98 Hz, 1H, Ar-H3), 7.85 (dd, *J* = 8.59, 1.98 Hz, 1H, Ar-H5), 7.23(d, *J* = 8.69 Hz, 1H, Ar-H6), 7.10 (m, 2H, imidazole-H), 4.03–4.17 (m, 4H, 2CH_2_), 2.48 (under DMSO, 2H, CH_2_), 1.94–2.08 (m, 2H, CH_2_), 1.18 (t, *J* = 7.08 Hz, 3H, CH_3_).

*Ethyl 4-[3-chloro-4-(1H-imidazol-2-yl)phenoxy]butanoate* (**16e**): greyish white solid (2.58 g, 50%), m.p. 168–172 °C. ^1^H-NMR (300 MHz, DMSO-*d*_6_) δ_H_ 7.68 (d, *J* = 8.69 Hz, 1H, Ar-H5), 7.05–7.19 (m, 3H, Ar-H2 and imidazole-H), 7.00 (dd, *J* = 8.69, 2.55 Hz, 1H, Ar-H6), 3.99–4.15 (m, 4H, 2CH_2_), 2.43–2.50 (under DMSO, 2H, CH_2_), 1.99 (quin, *J* = 6.75 Hz, 2H, CH_2_) 1.19 (t, *J* = 7.13 Hz, 3H, CH_3_).

### 3.16. General Procedure for the Synthesis of 2-[Di/monochloro-4-(1H-imidazol-2-yl)phenoxy]acetic/butyric Acids ***17a**–**e*** [43]

These compounds were prepared according to procedure described above for preparation of **7a**–**f**, starting with esters **16a**–**e** (3.2 mmol).

*2-[2,3-Dichloro-4-(1H-imidazol-2-yl)phenoxy]acetic acid* (**17a**): off-white solid (0.87 g, 82%), m.p. >300 °C (dec.). ^1^H-NMR (300 MHz, DMSO-*d*_6_) δ_H_ 15.08 (brs, 1H, OH), 7.81 (s, 1H, NH), 7.70 (d, *J* = 8.88 Hz, 1H, Ar-H6) 7.34 (d, *J* = 9.06 Hz, 1H, Ar-H5), 7.0–7.01 (m, 2H, imidazole-H), 5.00 (s, 2H,OCH_2_), LC/MS, *m*/*z* 286.9 (M + 1)^+^, 288.9 (M + 3)^+^, 291.1 (M + 5)^+^. Anal. Calcd for (C_11_H_8_Cl_2_N_2_O_3_): C, 46.02; H, 2.81; Cl, 24.70; N, 9.76; Found: C, 46.21; H, 3.02; Cl, 24.34; N, 9.49.

*[2-Chloro-4-(1H-imidazol-2-yl)phenoxy]acetic acid* (**17b**): off-white solid (1.2 g, 88.8%), m.p. >300 °C (dec.); ^1^H-NMR (300 MHz, DMSO-*d*_6_) δ_H_ 7.99 (d, *J* = 2.08 Hz, 1H, Ar-H3), 7.82 (dd, *J* = 8.69, 1.89 Hz, 1H, Ar-H5), 7.02–7.20 (m, 3H, Ar-H6 and imidazole-H), 4.85 (s, 2H, OCH_2_); LC/MS, *m/z* 251.0 (M^−^), 253.1 (M + 2)^−^.

*4-[2,3-Dichloro-4-(1H-imidazol-2-yl)phenoxy]butanoic acid* (**17c**): off-white solid (0.87 g, 82%), m.p. >300 °C (dec.); ^1^H-NMR (300 MHz, DMSO-*d*_6_) δ_H_ 12.23 (brs, 2H, NH and OH), 7.65 (d, *J* = 8.69 Hz, 1H, Ar-H6), 7.25 (d, *J* = 8.69 Hz, 1H, Ar-H5), 7.15 (s, 2H, imidazoline-H), 4.18 (t, *J* = 5.95 Hz, 2H, OCH_2_), 2.44 (t, *J* = 7.18 Hz, 2H, CH_2_), 1.93–2.08 (m, 2H, CH_2_); LC/MS, *m*/*z* 316.0 (M + 1)^+^, 318.0 (M + 3)^+^, 320.0 (M + 5)^+^.

*4-[2-Chloro-4-(1H-imidazol-2-yl)phenoxy]butanoic acid* (**17d**): pale brown solid (1.5 g, 83%), m.p. 219–220 °C; ^1^H-NMR (300 MHz, DMSO-*d*_6_) δ_H_ 12.47 (brs, 1H, OH), 12.20 (brs, 1H, NH), 7.98 (d, *J* = 1.89 Hz, 1H, Ar-H3), 7.85 (d, *J* = 8.59 Hz, 1H, Ar-H6), 7.23 (d, *J* = 8.69 Hz, 1H, Ar-H5), 7.11 (s, 2H, imidazole-H), 4.12 (t, *J* = 6.23 Hz, 2H, OCH_2_), 2.42 (t, *J* = 7.08 Hz, 2H, CH_2_), 1.95 (quin, *J =* 6.93 Hz, 2H, CH_2_); LC/MS (ESI), *m*/*z* 281.1 (M + 1)^+^, 283.1 (M + 3)^+^.

*4-[3-Chloro-4-(1H-imidazol-2-yl)phenoxy]butanoic acid* (**17e**): yellow solid (1.7 g, quantitative), m.p. 103–106 °C; ^1^H-NMR (300 MHz, DMSO-*d*_6_) δ_H_ 12.14 (brs, 1H, NH), 7.81 (d, *J* = 2.50 Hz, 1H, Ar-H2), 7.67 (d, *J* = 8.69 Hz, 1H, Ar-H5), 7.12 (d, *J* = 2.36 Hz, 2H, imidazole-H), 7.01 (dd, *J* = 8.73, 2.50 Hz, 1H, Ar-H6), 4.06 (t, *J* = 6.42 Hz, 2H, OCH_2_), 2.39 (t, *J* = 7.27 Hz, 2H, CH_2_), 1.95 (quin, *J* = 6.80 Hz, 2H, CH_2_); LC/MS, *m*/*z* 281.0 (M + 1)^+^, 283.1 (M + 3)^+^; Anal. Calcd for (C_13_H_13_ClN_2_O_3_): C, 55.62; H, 4.67; Cl, 12.63; N, 9.98; Found: C, 55.87; H, 5.01; Cl, 12.99; N, 9.88.

### 3.17. General Procedure for the Synthesis of 2,3-Di/monochloro-4-(3-hydroxypropoxy)benzaldehydes ***18a**–**c*** [42]

To a cooled stirred solution of the appropriate 4-hydroxybenzaldehyde (**1a**–**c**) (0.031 mol) and K_2_CO_3_ (0.157 mol), KI (0.003 mol) in DMF (60 mL), was added 3-chloro-1-propanol (6 g, 0.062 mol) at 5–10 °C. The mixture was stirred for 16 h at 70 °C, then left to attain room temperature. Ice and water were added and the crude product was extracted with DCM (3 × 100 mL). The combined organic extract was washed with water and brine, dried over anhydrous MgSO_4_. The crude compound was purified by flash chromatography using (EtOAc 1:hexane 4) to afford ethers **18a**–**c**.

*2,3-Dichloro-4-(3-hydroxypropoxy)benzaldehyde* (**18a**): pale yellow liquid (6.2 g, 79%); ^1^H-NMR (300 MHz, DMSO-*d*_6_) δ_H_10.19 (brs, 1H, CHO), 7.95 (brs, 1H, OH), 7.32 (d, *J =* 8.14 Hz, 1H, Ar-H6), 4.59–4.70 (m, 1H, Ar-H5), 4.28 (m, 2H, OCH_2_), 3.60 (q, *J* = 5.19 Hz, 2H, CH_2_), 1.84–2.02 (m, 2H, CH_2_).

*2-Chloro-4-(3-hydroxypropoxy)benzaldehyde* (**18b**): pale yellow liquid (9.5 g, 86.6%); ^1^H-NMR (300 MHz, DMSO-*d*_6_) δ_H_10.20 (s, 1H, CHO), 7.83 (d, *J* = 8.69 Hz, 1H, Ar-H6); 7.18 (d, *J* = 2.27 Hz, 1H, Ar-H3), 7.09 (dd, *J* = 8.73, 2.31 Hz, 1H, CH_5_), 4.61 (t, *J* = 5.15 Hz, 1H, OH), 4.19 (t, *J* = 6.37 Hz, 2H, OCH_2_), 3.55 (q, *J* = 5.89 Hz, 2H, CH_2_), 1.88 (quin, *J* = 6.26 Hz, 2H, CH_2_).

*3-Chloro-4-(3-hydroxypropoxy)benzaldehyde* (**18c**): pale yellow liquid (9.5 g, 86.6%). ^1^H-NMR (300 MHz, DMSO-*d*_6_) δ_H_ 9.85 (s, 1H, CHO), 7.70–8.08 (m, 2H, Ar-H2, Ar-H6), 7.34 (d, *J* = 8.50 Hz, 1H, Ar-H5), 4.55–4.69 (brs, 1H, OH), 4.24 (t, *J* = 6.18 Hz, 2H, OCH_2_), 3.53–3.68 (m, 2H, CH_2_), 1.92 (quin, *J* = 6.09 Hz, 2H, CH_2_).

### 3.18. General Procedure for the Synthesis of 3-[Di/monochloro-4-(4,5-dihydro-1H-imidazol-2-yl)phenoxy]propan-1-ols ***19a**–**c*** [44]

These intermediates were prepared according to procedure described above for the preparation of **15a**–**e** starting with the appropriate 4-(3-hydroxypropoxy)benzaldehyde derivatives **18a**–**c** (0.016 mol).

*3-[2,3-Dichloro-4-(4,5-dihydro-1H-imidazol-2-yl)phenoxy]propan-1-ol* (**19a**): pale yellow liquid (4 g, 86%); ^1^H-NMR (300 MHz, DMSO-*d*_6_) δ_H_ 7.48 (d, *J* = 8.69 Hz, 1H, Ar-H6), 7.20 (d, *J* = 8.78 Hz, 1H, Ar-H5), 6.73 (brs, 1H, NH), 4.62 (brs, 1H, OH), 4.20 (t, *J* = 6.00 Hz, 2H, imidazoline-H), 3.78 (t, *J* = 8.31 Hz, 2H, imidazoline-H), 3.59 (brs, 2H, OCH_2_), 3.44 (t, *J* = 7.77 Hz, 2H, CH_2_),1.93 (quin, *J* = 6.72 Hz, 2H, CH_2_).

*3-[2-Chloro-4-(4,5-dihydro-1H-imidazol-2-yl)phenoxy]propan-1-ol* (**19b**): pale yellow solid (7.74 g, 69.2%), m.p. 88–91 °C. ^1^H-NMR (300 MHz, DMSO-*d*_6_) δ_H_ 7.85 (d, *J* = 1.98 Hz, 1H, Ar-H3), 7.75 (dd, *J* = 8.59, 1.98 Hz, 1H, Ar-H5) 7.20 (d, *J* = 8.69 Hz, 1H, Ar-H6), 6.93 (brs, 1H, NH), 4.61 (brs, 1H, OH), 4.17 (t, *J* = 6.18 Hz, 2H, imidazoline-H), 3.75 (brs, 2H, imidazoline-H), 3.59 (t, *J* = 6.00 Hz, 2H, OCH_2_), 3.41 (brs, 2H, CH_2_), 1.90 (quin, *J* = 6.18 Hz, 2H, CH_2_).

*3-[3-Chloro-4-(4,5-dihydro-1H-imidazol-2-yl)phenoxy]propan-1-ol* (**19c**): pale yellow liquid (10.3 g, 96.5%); ^1^H-NMR (300 MHz, DMSO-*d*_6_) δ_H_ 7.52 (d, *J* = 8.69 Hz, 1H, Ar-H5), 7.05 (d, *J* = 2.27 Hz, 1H, Ar-H2), 6.94 (dd, *J* = 8.64, 2.41 Hz, 1H, Ar-H6), 6.63 (brs, 1H, NH), 4.59 (d, *J* = 4.44 Hz, 1H, OH) 4.09 (t, *J* = 6.33 Hz, 2H, imidazoline-H), 3.54 (t, *J* = 6.18 Hz, 2H, imidazoline-H), 3.48 (t, *J* = 6.00 Hz, 2H, OCH_2_), 3.41 (brs, 2H, CH_2_), 1.85 (quin, *J* = 6.28 Hz, 2H, CH_2_).

### 3.19. General Procedure for the Synthesis of 3-[Di/monochloro-4-(1H-imidazol-2-yl)phenoxy]propan-1-ols ***20a**–**c*** [44]

These intermediates were prepared following the same procedure described above for the preparation of **16a**–**c** starting with the appropriate **19a**–**c** (0.027 mol).

*3-[2,3-Dichloro-4-(1H-imidazol-2-yl)phenoxy]propan-1-ol* (**20a**): pale yellow liquid (3.8 g, 50 %); ^1^H-NMR (300 MHz, DMSO-*d*_6_) δ_H_ 12.29 (brs, 1H, NH) 7.66 (d, *J* = 8.78 Hz, 1H, Ar-H6), 7.26 (d, *J* = 8.88 Hz, 1H, Ar-H5), 7.15 (m, 2H, imidazole-H), 4.62 (brs, 1H, OH), 4.22 (t, *J* = 6.04 Hz, 2H, OCH_2_), 3.53–3.61 (m, 2H, CH_2_), 1.92 (t, *J* = 6.09 Hz, 2H, CH_2_).

*3-[2-Chloro-4-(1H-imidazol-2-yl)phenoxy]propan-1-ol* (**20b**): pale yellow liquid (3.2 g, 42%); ^1^H-NMR (300 MHz, DMSO-*d*_6_) δ_H_ 12.43 (brs, 1H, NH), 7.98 (d, *J* = 1.98 Hz, 1H, Ar-H3), 7.85 (dd, *J* = 8.59, 1.98 Hz, 1H, Ar-H5), 7.24 (d, *J* = 8.69 Hz, 1H, Ar-H6), 7.10 (brs, 2H, imidazole-H), 4.59 (t, *J* = 5.10 Hz, 1H, OH), 4.17 (t, *J* = 6.23 Hz, 2H, OCH_2_) 3.60 (q, *J* = 5.92 Hz, 2H, CH_2_), 1.90 (quin, *J* = 6.1 Hz, 2H, CH_2_).

*3-[3-Chloro-4-(1H-imidazol-2-yl)phenoxy]propan-1-ol* (**20c**): wine red liquid (7.1 g, 70%); ^1^H-NMR (300 MHz, DMSO-*d*_6_) δ_H_ 12.16 (brs, 1H, NH), 7.93 (d, *J* = 1.98 Hz, 1H, Ar-H2), 7.67 (d, *J* = 8.69 Hz, 1H, Ar-H5), 7.07–7.17 (m, 2H, imidazole-H), 7.01 (dd, *J* = 8.69, 2.36 Hz, 1H, Ar-H6), 4.60 (brs, 1H, OH), 4.11 (t, *J* = 6.28 Hz, 2H, OCH_2_), 3.56 (d, *J* = 4.25 Hz, 2H, CH_2_), 1.87 (quin, *J* = 6.2 Hz, 2H, CH_2_).

### 3.20. General Procedure for the Synthesis of 3-[2,3-Di/monochloro-4-(1H-imidazol-2-yl)phenoxy]propanoic Acids ***21a–c*** [42]

To a stirred solution of the appropriate alcohol **20a**–**c** (12.8 mmol) in acetone (40 mL), a solution of chromium trioxide (68 mmol) in H_2_O (50 mL) and AcOH (6 mL) was added while keeping the temperature between 5–10 °C. The mixture was stirred for 30 min at the same temperature then warmed up to room temperature for 16 h. The reaction was monitored by LC-MS till complete disappearance of the starting material. Then the reaction mixture was treated with NaHCO_3_ and the pH was adjusted to 8. The mixture was washed with DCM (3 × 50 mL) and the aqueous layer was concentrated under reduced pressure to 50 mL. Again, the pH was adjusted to pH 5 (1.5 N HCl). The precipitated solid product was collected by filtration, washed with water and dried. Purification by crystallization from MeOH gave the required products (**21a**–**c**).

*3-[2,3-Dichloro-4-(1H-imidazol-2-yl)phenoxy]propanoic* *acids* (**21a**): off-white solid (0.73 g, 18%), m.p. 193–194 °C. IR (KBr, ν_max_ cm^−1^): 3585(NH), 3100 (OH), 1620 (CO), 1294, 1023 (C-O-C); ^1^H-NMR (300 MHz, DMSO-*d*_6_) δ_H_ 13.1(brs, 1H, OH), 12.4 (brs, 1H, NH), 7.64 (d, *J* = 8.50 Hz, 1H, Ar-H6), 7.27 (d, *J* = 8.69 Hz, 1H, Ar-H5), 7.15 (s, 2H, imidazole-H), 4.32 (t, *J =* 8.2 Hz, 2H, OCH_2_), 2.67 (t, *J =* 8.2 Hz, 2H, CH_2_); LC/MS: *m*/*z* 300.0 (M)^−^, 302.0 (M + 2)^−^, 304.0 (M + 4)^−^.

*3-[2-Chloro-4-(1H-imidazol-2-yl)phenoxy]propanoic acid* (**21b**): off-white solid (0.23 g, 44%), m.p. 210–213 °C. IR (KBr, ν_max_ cm^−1^): 3443 (NH), 3142 (OH), 1687 (CO), 1269, 1065 (C-O-C); ^1^H-NMR (300 MHz, DMSO-*d*_6_) δ_H_ 12.35 (brs, 1H, OH), 7.97 (brs, 1H, NH), 7.86 (d, *J* = 8.59 Hz, 1H, Ar-H5), 7.27 (d, *J* = 8.40 Hz, 1H, Ar-H6), 7.11 (m, 3H, Ar-H3 and imidazole-H), 4.30 (t, *J =* 7.1 Hz, 2H, CH_2_), 2.74 (t, *J =* 6.2 Hz, 2H, CH_2_); LC/MS: *m*/*z* 267.0 (M + 1), 269.1 (M + 2); Anal. Calcd for (C_13_H_11_ClN_2_O_3_): C, 54.05; H, 4.16; Cl, 13.29; N, 10.50; Found: C, 53.79; H, 3.82; Cl, 13.61; N, 10.55.

*3-[3-Chloro-4-(1H-imidazol-2-yl)phenoxy]propanoic acid* (**21c**): off-white solid (0.6 g, 9.5%), m.p. 208–210 °C. IR (KBr, ν_max_ cm^−1^): 3400 (NH), 3139 (OH), 1694 (CO), 1296, 1092 (C-O-C); ^1^H-NMR (300 MHz, DMSO-*d*_6_) δ_H_ 13.0, 12.21 (two brs, each 1H, OH and NH), 7.67 (d, *J* = 8.69 Hz, 1H, Ar-H5), 7.12 (m, 3H, Ar-H2 and imidazole-H), 7.01 (d, *J* = 7.08 Hz, 1H, Ar-H6), 4.24 (t, *J* = 5.17 Hz, 2H, OCH_2_), 2.70 (t, *J* = 5.43 Hz, 2H, CH_2_); LC/MS: *m*/*z* 267.0 (M + 1)^−^, 269.0 (M + 3)^−^.

### 3.21. RBC Morphological Antisickling Studies

KAUS-4, KAUS-23 and KAUS-24, and clofibrate were tested for their effect on sickle RBC morphology as previously reported [18,19,22]. Briefly sickle RBCs were incubated under air in the absence or presence of 2 mM concentration of test compound (solubilized in DMSO) at room temperature for 1 h. Following, the suspension was incubated under hypoxic condition (4% oxygen/96% nitrogen) at 37 °C for 3 h. The suspension was fixed with 2% glutaraldehyde solution without exposure to air and then subjected to microscopic morphological analysis. 

### 3.22. Oxygen Equilibrium Curve Studies

Normal blood samples (hematocrit 22%) in the absence (control) or presence of 2 mM concentration of the test KAUS compounds (solubilized in DMSO) were incubated at 37 °C for 1.5 h and then subjected to OEC analysis using tonometry as previously described [18,19,22]. Briefly, the compound-treated blood samples is incubated in IL 237 tonometers (Instrumentation Laboratories, Inc. Lexington, MA, USA) for approximately 10 min at 37 °C, and allowed to equilibrate at oxygen tensions 7, 20, 40 and 60 mmHg. The samples were then aspirated into an ABL 700 Automated Blood Gas Analyzer (Radiometer) to determine the pH, partial pressure of CO_2_ (pCO_2_), partial pressure of oxygen (pO_2_), and Hb oxygen saturation values (SO_2_). The measured values of pO_2_ (mmHg) and SO_2_ at each pO_2_ value were then subjected to a non-linear regression analysis using the program Scientist (Micromath, Salt Lake City, UT, USA) to estimate P_50_ and Hill coefficient values (n_50_). Clofibrate was tested as a positive control, while DMSO was tested as negative control.

### 3.23. Crystallization, Data Collection and Structure Determination of Deoxygenated Hb in Complex with KAUS-23

Freshly prepared solution of KAUS-23 in DMSO was incubated with deoxygenated Hb (40 mg/mL) for 30 min at Hb tetramer:KAUS-23 molar ratio of 1:10 at room temperature and then crystallized with 3.2 M sulfate/phosphate precipitant, pH 6.8 using the batch method as previously described [18,19,22]. Diffraction data was collected at 100 K on an R-axis IV++ image plate detector using CuKα X-ray (λ = 1.5417) from a Rigaku Micro-Max™ -007 X-ray source equipped with Varimax confocal optics operating at 40 kV and 20 mA (Rigaku, The Woodlands, TX, USA). Crystals were cryoprotected with 15% glycerol. The dataset was processed with the d*trek software (v9.9.9.7, Rigaku Corporation, Tokyo, Japan) and the CCP4 suite of programs [45]. 

The isomorphous native human deoxygenated Hb tetramer structure (PDB code 2DN2) was used as the starting model to refine the structure, using both Phenix and CNS refinement programs [46,47]. Model building and correction were carried out using COOT [48,49]. A round of refinement showed a bound KAUS-23 molecule at the central water cavity. The symmetry-related site was poorly defined. Therefore, only one KAUS-23 molecule was built into the model and refined. Also, included in the final model are two molecules of sulfate ions, and 400 water molecules. The structure refined to a final Rfactor/Rfree of 19.68/24.92% at a resolution of 2.15 Å. The atomic coordinate and structure factor files have been deposited in the RCSB Protein Data Bank with accession codes 5KDQ. Detailed crystallographic and structural analysis parameters are reported in Table 3.

## 4. Conclusions 

Aryloxyalkanoic acids that bind non-covalently to the αTrp14 hydrophobic pocket of Hb have been shown to exhibit antisickling activities, while those that bind to the central water cavity of the protein in most instances showed the opposite effect [25,26,27,28,29]. The novel KAUS molecules were designed to have an imidazole pharmacophore, which was expected to increase hydrophobic interactions at the αTrp14 binding site and result in enhanced antisickling activity. However, our structural modifications did not work as anticipated, as these compounds appear to bind to the central water cavity instead, stabilizing the T-state Hb and resulting mostly in a decrease in Hb affinity for oxygen. Considering that compounds that decrease Hb affinity for oxygen and enhance O2 delivery to tissues have been studied to treat hypoxic- or ischemic-related diseases [50,51,52,53,54,55,56,57], this series of compounds may potentially be useful. For example, RSR-13 and several of its analogs that bind to the central water cavity of Hb and significantly decrease Hb affinity for oxygen (∆P_50_ of 20–30 mmHg at 2 mM) have been the subject of investigation for stroke, wound healing, and as a radiation enhancer in the radiotherapy of hypoxic tumors, adjuncts for Hb based oxygen carriers, nitric oxide, carbon monoxide and other non-oxygen therapeutic gases [50,51,52,53,54,55,56,57]. These compounds were designed based on clofibrate atomic interaction with Hb [2,30,31,32,37,57,58]. Like clofibrate, the KAUS compounds bind to the central water cavity of Hb and weakly decrease Hb affinity for oxygen by stabilizing the T-state. The structure provides direction and approach for designing a new class of potent Hb allosteric compounds that decrease Hb affinity for oxygen. We note that the alkanoic acid moiety of KAUS-23 is close to the side-chains of αTyr140, αPro95, αVal96, βTrp37 and αLys99; residues that are known to move significantly during the T↔ R transition. By incorporating another phenyl group between the methoxyphenyl and the alkanoic acid not only would allow increased hydrophobic interactions with these residues, but also extend the carboxylate to potentially form salt-bridge/hydrogen-bond interactions with αLys99. We therefore anticipate these interactions to lock the residues in the T-state conformation and lead to further stabilization of the T-state with a concomitant decrease in Hb affinity for oxygen. 

Besides aryloxyalkanoic acids, non-acidic halogenated benzene derivatives or toluene are also known to bind to the αTrp14 hydrophobic pocket [26,33,59]. However, unlike the aryloxyalkanoic acids, the halogenated benzenes or toluene bind exclusively to the αTrp14 site. It is not obvious what is directing the KAUS compounds into the central water cavity because modelling KAUS-23 at the αTrp14 binding pocket (Figure 2b) suggested a more favorable binding when compared to its actual binding in the central water cavity (see Supplementary Material). We speculate that like the large number of Hb effectors with carboxylate moiety [29,30,31,32], the central water cavity which is lined with several positive residues is acting like a sink to attract these carboxylate compounds. As part of our future investigations, we plan to modify or eliminate the acidic moieties from the KAUS compounds, which would likely lead to the desired interactions at the αTrp14 site, and result in corresponding antisickling activity.

## Figures and Tables

**Table 1:**
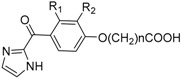
The Effect of Compounds **7a–i** on Hb Affinity for Oxygen Using Normal Whole Blood ^a^.

Comp.	Name	R_1_	R_2_	N	Mean P_50_^b^	∆P_50_^c^
**Ctr**	DMSO ^d^				38.3 ± 0.0	0.0
**Ctr**	Clofibrate				40.3 ± 0.1	2.0 ± 0.1
**7a**	KAUS-1	Cl	Cl	1	38.3 ± 0.4	0.0 ± 0.4
**7b**	KAUS-4	H	Cl	1	37.5 ± 0.3	−0.8 ± 0.3
**7c**	KAUS-7	Cl	H	1	39.3 ± 0.6	1.0 ± 0.6
**7d**	KAUS-3	Cl	Cl	3	38.6 ± 0.1	0.3 ± 0.1
**7e**	KAUS-6	H	Cl	3	38.6 ± 0.6	0.3 ± 0.6
**7f**	KAUS-9	Cl	H	3	38.2 ± 0.5	−0.1 ± 0.5
**7g**	KAUS-2	Cl	Cl	2	38.6 ± 0.1	0.3 ± 0.1
**7h**	KAUS-5	H	Cl	2	39.2 ± 0.4	0.9 ± 0.4
**7i**	KAUS-8	Cl	H	2	38.6 ± 0.4	0.3 ± 0.4

^a^ Results are the means of two or three measurements. ^b^ P_50_ is the oxygen pressure in mmHg at which normal RBC (22% hematocrit) is 50% saturated with oxygen. ^c^ ΔP_50_ (mm Hg) is P_50_ of compound treated cells − P_50_ of control. ^d^ The final concentration of DMSO was <2% in all samples.

**Table 2:**
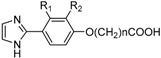
The Effect of Compounds (**13**, **17a**–**e**, **21a**–**c**) on Hb Affinity for Oxygen Using Normal Whole Blood ^a^.

Comp.	Name	R_1_	R_2_	N	Mean P_50_^b^	∆P_50_^c^
**Ctr**	DMSO ^d^				38.3 ± 0.0	0.0
**13**	KAUS-25	Cl	H	1	39.7 ± 0.3	1.4 ± 0.3
**17a**	KAUS-19	Cl	Cl	1	39.2 ± 0.2	0.9 ± 0.2
**17b**	KAUS-22	H	Cl	1	41.3 ± 0.05	2.9 ± 0.05
**21c**	KAUS-26	Cl	H	2	39.7 ± 0.1	1.4 ± 0.1
**21a**	KAUS-20	Cl	Cl	2	38.9 ± 0.3	0.7 ± 0.3
**21b**	KAUS-23	H	Cl	2	40.8 ± 0.02	2.5 ± 0.02
**17e**	KAUS-27	Cl	H	3	39.1 ± 0.3	0.8 ± 0.3
**17c**	KAUS-21	Cl	Cl	3	39.6 ± 0.6	1.3 ± 0.6
**17d**	KAUS-24	H	Cl	3	41.7 ± 0.3	3.4 ± 0.3

^a^ The results are the means of two or three measurements. ^b^ P_50_ is the oxygen pressure at which normal RBC (22% hematocrit) is 50% saturated with oxygen. ^c^ ΔP_50_ is P_50_ of compound treated cells—P_50_ of control. ^d^ The final concentration of DMSO was <2% in all samples.

**Table 3:** Data collection and refinement statistics of deoxygenated Hb in complex with KAUS-23. Numbers in parentheses are for the highest resolution shell.

Data	KAUS-23
**Space group**	P2_1_
**Unit-cell *a,b,c* (Å)**	62.2, 80.8, 53.4, 90.0, 99.2, 90.0
**Resolution (Å)**	29.64–2.15 (2.23–2.15)
**Unique reflections**	27383
**Redundancy**	6.75
**Completeness (%)**	96.2 (94.1)
**Average I/σ(I)**	21.0 (8.4)
**R_merge_ (%) ^a^**	6.4 (14.6)
**Refinement ^b^**	
**Resolution (Å)**	29.64–2.15 (2.23–2.15)
**No. of reflections**	27358 (2521)
**R_work_ (%)**	19.24 (24.73)
**R_free_ (%)**	25.71 (35.69)
**R.m.s.d. bonds (Å)**	0.007
**R.m.s.d. angles (°)**	1.28
**Dihedral angles**	
**Most favored (%)**	95.6
**Allowed (%)**	3.7
**Average B (Å^2^) / atoms**	
**All atoms**	29.4
**Protein**	29.0
**Hemes**	27.2
**KAUS-23**	40.7
**Phosphate**	54.6
**Water**	34.7
**PDB ID code**	5KDQ

^a^ *R*_merge_ = Σ*_hkl_*Σ_i_|*I*_i_(*hkl*) – <*I*(*hkl*)>|/Σ*_hkl_*Σ_i_*I*_i_(*hkl*)*.* ^b^ R_free_ was calculated from 5% randomly selected reflection for cross-validation. All other measured reflections were used during refinement.

## Supplementary Materials

Supplementary materials can be accessed at: http://www.mdpi.com/1420-3049/21/8/1057/s1.
