# Supplementary material for: Aryloxyalkanoic Acids as Non-Covalent Modifiers of the Allosteric Properties of Hemoglobin
Source: Molecules. 2016 Aug 13;21(8):1057. doi: 10.3390/molecules21081057 (PMC5453642; doi:10.3390/molecules21081057)
Supplement: Supplementary file 1 [file molecules-21-01057-s001.pdf]

Supplementary Materials: Aryloxyalkanoic Acids as Non-Covalent Modifiers of the Allosteric Properties of Hemoglobin

Abdelsattar M. Omar, Mona A. Mahran, Mohini S. Ghatge, Faida H. A. Bamane, Mostafa H. Ahmed, Moustafa E. El-Araby, Osheiza Abdulmalik and Martin K. Safo

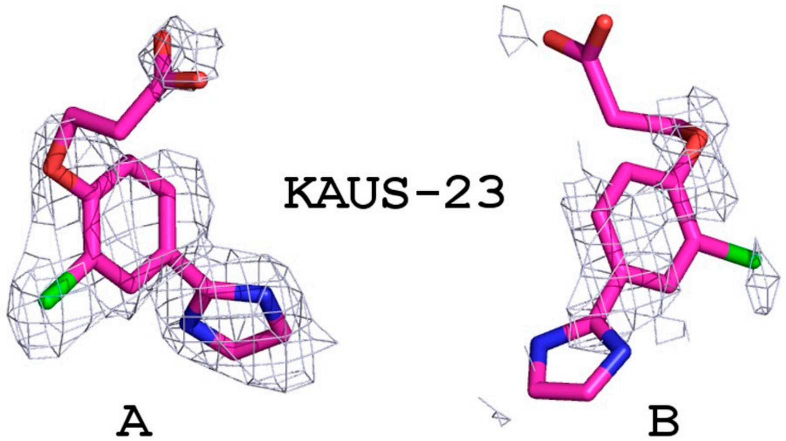

Figure S1. Final 2Fo-Fc refined electron density map contoured at 0.7σ. Note that while the binding site (**A**) was actually fitted with KAUS-23 and refined, the symmetry-related site (**B**) was fitted with water molecules during the refinement due to lack of well-defined density. Carbon atoms are shown in magenta, Oxygen atoms in red, Nitrogen in blue and Chlorine in green. Image was created by PyMOL (Schrödinger, LLC, New York, NY, USA).

Molecular Modelling Studies:

The crystal structure of the deoxy form of hemoglobin (PDB ID: 2DN2) was used for the docking studies. First, hydrogen atoms were added and minimized while keeping all heavy atoms as aggregates using SYBYL-X 2.1 (Tripos LPMO, Princeton, NJ, USA). KAUS-23 was modeled in SYBYL-X 2.1 (Tripos LPMO, USA) and docked into the structure of deoxyhemoglobin using GOLD version 5.3 (The Cambridge Crystallographic Data Centre Inc., Cambridge, UK). The binding site was defined as 10 Å around the center of the cavity surrounded by residues αLys11, αTrp14, αAla21 and αThr67 of α1-chain. Since the conformation of αTrp14A is changed dramatically upon binding to toluene (PDB ID: 1R1X) it was kept flexible during the docking. The genetic algorithm search efficiency was set to the maximum (200%) to do an exhaustive search for the optimal pose. The default settings were used for the rest of the parameters in GOLD. Finally, each docked pose was scored using HINT (eduSoft LC, Richmond, VA, USA) for final pose selection. The predicted pose (Figure 2b) with the most favorable binding gave a HINT score of ~1000, which compares with a HINT score of ~120 for the crystallographically bound KAUS-23 in the central water cavity.
